# Synthesis of zeolite from industrial wastes: a review on characterization and heavy metal and dye removal

**DOI:** 10.1007/s11356-024-33863-0

**Published:** 2024-06-11

**Authors:** Sena Eren, Feride N. Türk, Hasan Arslanoğlu

**Affiliations:** 1 Canakkale Onsekiz Mart University, Faculty of Engineering, Department of Chemical Engineering, Çanakkale, Turkey; 2 Çankırı Karatekin University, Central Research Laboratory Application and Research Center, Çankırı, Turkey

**Keywords:** Heavy metal and dye removal, Industrial waste, Synthesis method, ^29Si^NMR-^27Al^NMR

## Abstract

Increasing world population, urbanization, and industrialization have led to an increase in demand in production and consumption, resulting in an increase in industrial solid wastes and pollutant levels in water. These two main consequences have become global problems. The high Si and Al content of solid wastes suggests that they can be used as raw materials for the synthesis of zeolites. In this context, when the literature studies conducted to obtain synthetic zeolites are evaluated, it is seen that hydrothermal synthesis method is generally used. In order to improve the performance of the hydrothermal synthesis method in terms of energy cost, synthesis time, and even product quality, additional methods such as alkaline fusion, ultrasonic effect, and microwave support have been developed. The zeolites synthesized by different techniques exhibit superior properties such as high surface area and well-defined pore sizes, thermal stability, high cation exchange capacity, high regeneration ability, and catalytic activity. Due to these specific properties, zeolites are recognized as one of the most effective methods for the removal of pollutants. The toxic properties of heavy metals and dyes in water and their carcinogenic effects in long-term exposure pose a serious risk to living organisms. Therefore, they should be treated at specified levels before discharge to the environment. In this review study, processes including different methods developed for the production of zeolites from industrial solid wastes were evaluated. Studies using synthetic zeolites for the removal of high levels of health and environmental risks such as heavy metals and dyes are reviewed. In addition, EPMA, SEM, EDX, FTIR, BET, AFM, and ^29^Si and ^27^Al NMR techniques, which are characterization methods of synthetic zeolites, are presented and the cation exchange capacity, thermodynamics of adsorption, effect of temperature, and pH are investigated. It is expected that energy consumption can be reduced by large-scale applications of alternative techniques developed for zeolite synthesis and their introduction into the industry. It is envisaged that zeolites synthesized by utilizing wastes will be effective in obtaining a green technology. The use of synthesized zeolites in a wide variety of applications, especially in environmental problems, holds great promise.

**Graphical Abstract:**

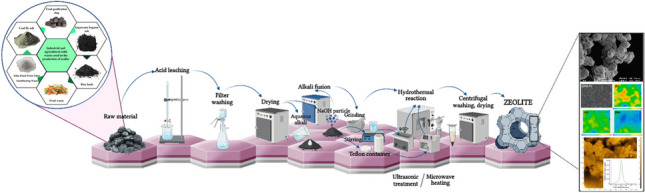

## Introduction

Water, which is an indispensable part of life, is under the negative effects of industrialization and modernization (Gul et al. 2022). There is a great increase in the environmental pollution problem due to the mixing of various pollutants into water due to increases in industrial production. The need for drinking water is increasing due to population growth in developing countries, so studies to prevent water pollution are of great importance. Heavy metals and dyes, which have a large mass among industrial wastes, are components that cause serious negative effects on human health (Batubara et al. 2023). There are many different techniques such as chemical precipitation, ion exchange, oxidation, membrane separation, coagulation and filtration, electrochemical processes, and adsorption techniques to purify heavy metals and dyes from wastewater (Iwuozor et al. 2021). Among these techniques, the adsorption method is a widely used method in the removal of heavy metals and dyes with its advantages such as flexibility, ease of use and design, and low installation costs (Iwuozor et al. 2021).

In the adsorption technique, activated carbons, metal organic cages (MOF) (Maraddi et al. 2024), graphene oxides (Adel et al. 2022), biosorbents (Li et al. 2024; Tokay & Akpınar 2021), biochars (Liu et al. 2023), nanoparticles, polymers, and clays are used (Abbou et al. 2023). Different adsorbents are used, such as minerals (Khoshraftar et al. 2023), composites, and zeolites (Murukutti & Jena 2022; Supelano et al. 2020).

Zeolites are used in water purification; removal of hazardous metal ions; removal of organic pollutants from wastewater; removal of ammonia, nitrogen, and phosphates from wastewater; applications in gas treatment; adsorption of volatile organic compounds (VOC); CO_2_ adsorption; adsorption of other gases; applications in catalysis; application in agriculture; and biomedical applications. It has various uses (Cao et al. 2023). Zeolites are materials that are naturally occurring and can be synthesized in the laboratory. Today, although the search for silica-alumina-containing raw materials suitable for the structure for the production of synthetic zeolites continues, as a result of literature research, it has been observed that solid wastes such as coal fly ash (Zhou et al. 2023), coal gangue (Yang et al. 2023), rice husk (Jin et al. 2023), and coal gasification slag (Cao et al. 2023) are used (Batubara et al. 2023). Zeolites are commonly synthesized by conventional hydrothermal synthesis method, alkaline fusion–assisted hydrothermal synthesis method (Ayele et al. 2016), microwave-assisted synthesis method (Truttim et al. 2023), and ultrasonic hydrothermal synthesis (Yin et al. 2023) methods.

Although the hydrothermal synthesis method requires a simple process, it has disadvantages such as long synthesis time and high energy consumption. For this reason, the method can be improved by applying additional processes such as alkaline fusion, ultrasonic effect, and microwave support (Tauanov et al. 2018). Different techniques are available for structural characterization of synthetic zeolites. Frequently, the physical properties, thermal behavior, and porosity of zeolites, as well as their mineral composition, are determined by methods such as thermogravimetry, N_2_ adsorption/desorption, optical microscopic methods, XRF, XRD, SEM/EDS, and EPMA/WDS (Khosravi et al. 2021).

This article first introduces the physicochemical properties of potential Si- and Al-containing industrial wastes for zeolite synthesis. Then, it explains the synthesis methods of zeolites and investigates the synthesis parameters of the studies in the literature. It compiles the studies on the subject on the recommendation of the use of zeolites in the removal of heavy metals and dyestuffs in wastewater. It compares and examines the studies on heavy metal and dye removal with zeolites synthesized from different raw materials. It explains the potential application areas of zeolites, the advantages they offer for future studies, and suitable analysis methods for the structural characterization of synthetic zeolites. This article, which can provide an in-depth understanding of zeolites, may also offer new ideas for studies on waste recovery and wastewater treatment.

## Zeolite

**Fig. 1:**
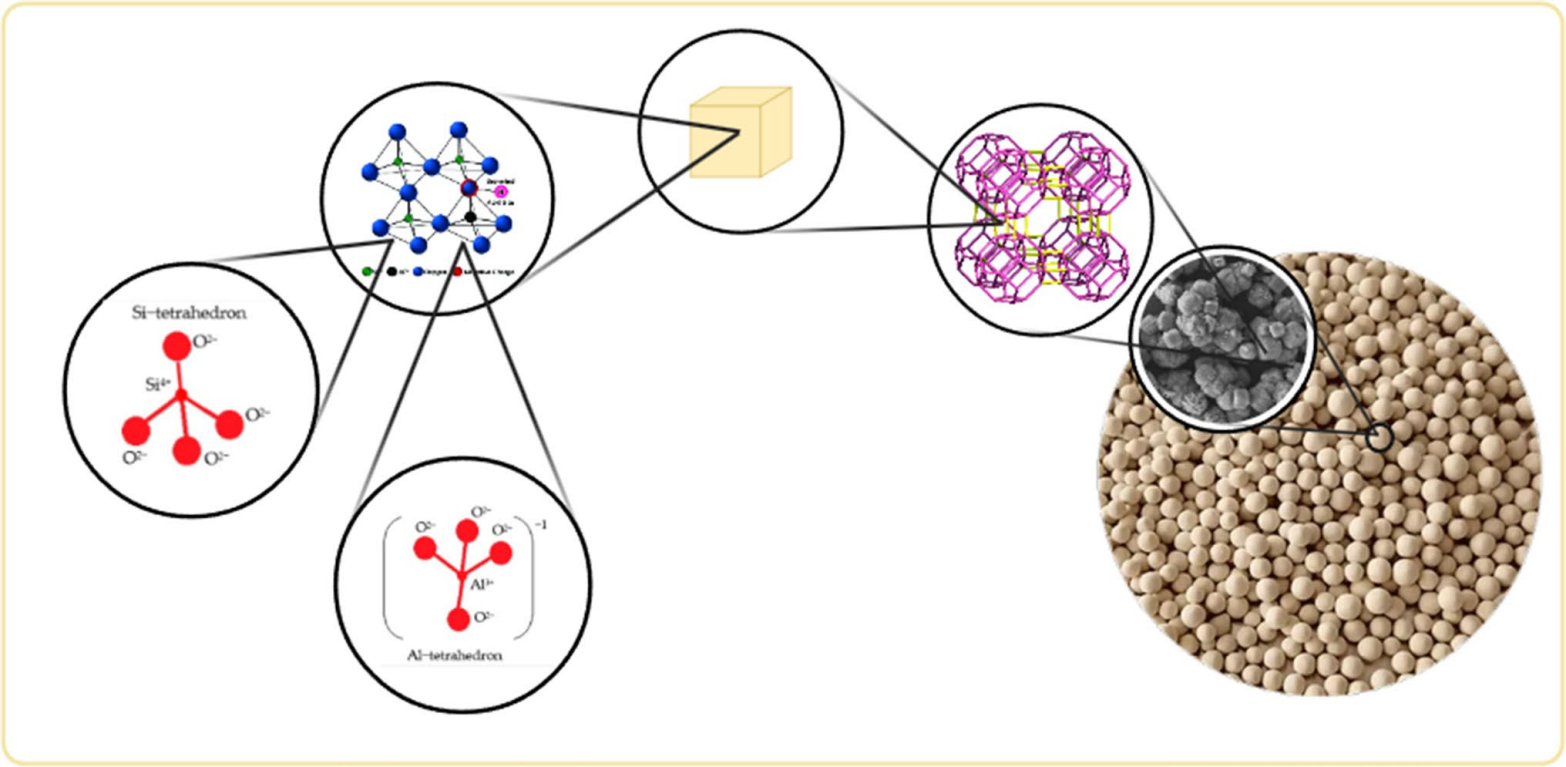
Structural representation of zeolites

Zeolite is a crystalline aluminosilicate consisting of alkali or alkaline earth metals, with a three-dimensional microporous structure, generally formed by sharing an oxygen atom between [SiO_4_]^4−^ (silicate) and [AlO_4_]^5−^ (aluminate) tetrahedrons. Although zeolite can be found naturally, it is a material with 200 different synthetic types synthesized depending on the change in the Si/Al ratio in the structure. The structural representation of zeolites is given in Fig. 1 (Cao et al. 2023).

Due to their properties such as well-defined pore sizes, compositional adjustability, and thermal stability, these materials, which have been used commercially in many areas since the 1950s, have increased their use in industrial and domestic applications today (Pérez-Botella et al. 2022).

**Fig. 2:**
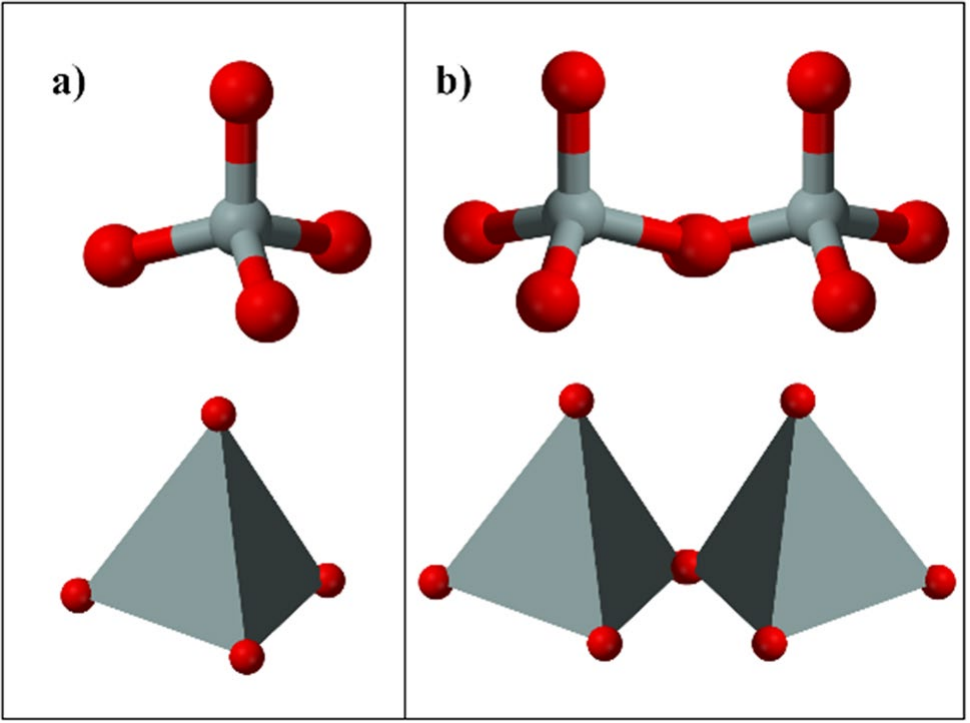
**a** TO_4_ (where T is the elements aluminum or Si). **b** Two tetrahedral structures sharing the oxygen atom (Bensafi et al. 2023)

It was first proposed in 1956 by Swedish mineralogist Alex F. Cronstedt, who observed that foam formed on the surface of a mineral sample by heating it. According to the appearance of the mineral, he defined the name as “zeolite,” which consists of the Greek words zein “to boil” and lithos “stone.” The first industrial success of zeolite material in subsequent times was its use as water softeners for laundry compositions, based on its ion exchange properties. This practice still continues today (Pérez-Botella et al. 2022). While research on the zeolite mineral was continuing, in 1840, it was discovered by Damour that the crystals of zeolite could be reversibly hydrated and dried without changing their transparency or morphology (Khaleque et al. 2020). The first zeolite synthesized with tetraalkylammonium cations by R. M. Barrer (1970) remains the most efficient strategy to obtain new zeolitic materials to this day. Inspired by McBain and Barrer’s work on molecular sieving, Robert M. Milton attempted an adsorption method to separate N_2_ from O_2_ instead of conventional cryogenic distillation using chabazite as the adsorbent. While trying to obtain this zeolite, changing the synthesis conditions such as lowering the temperature to 25–150 °C, using more reactive silica sources, and using a more alkaline environment enabled the rapid preparation of zeolites A and X, along with other new zeolite materials. After Donald W. Breck joined Milton’s group in 1951, zeolite Y was discovered in 1954. This zeolite type is isostructural with zeolite X and has lower Al content (Pérez-Botella et al. 2022). In the following years, with the discovery of many new and modified zeolites, applications in the fields of adsorption, ion exchange, and catalysis have been intensified. Zeolites have crystalline aluminosilicate structures composed of negatively charged inorganic frameworks. Framework structures are characterized based on pore diameters, geometry of crystal internal channels, and exchangeable cations. The zeolitic framework consists of a three-dimensional TO_4_ (here T aluminum or Si elements) tetrahedral structure by sharing an oxygen atom (Bensafi et al. 2023) (Fig. 2).

Zeolitic cages are formed by connecting pore openings varying in the range of approximately 0.3–1.0 nm in tetrahedral structures. The negative charges in the cage structure are neutralized by cations, creating a neutral structure. Their general chemical composition is “Ma/b[(AlO_2_)a(SiO_2_)y].cH_2_O” (M is the alkali metal or alkaline earth metal cation, b is the earth metal cation, c is the amount of crystallized water, a and y are [SiO_4_] in a unit cell of the zeolite. It is expressed as the total number of]^4−^ and [AlO_4_]^5−^ tetrahedral (Table 1.).

**Table 1:**
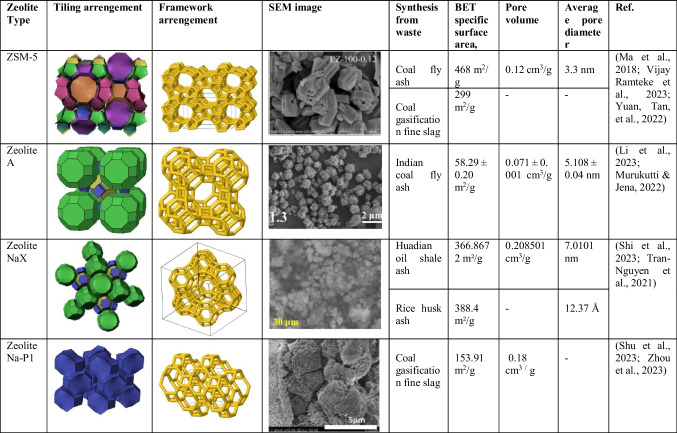
Commonly synthesized zeolite types and their properties

## Industrial wastes from Si and Al used in zeolite synthesis

**Fig. 3:**
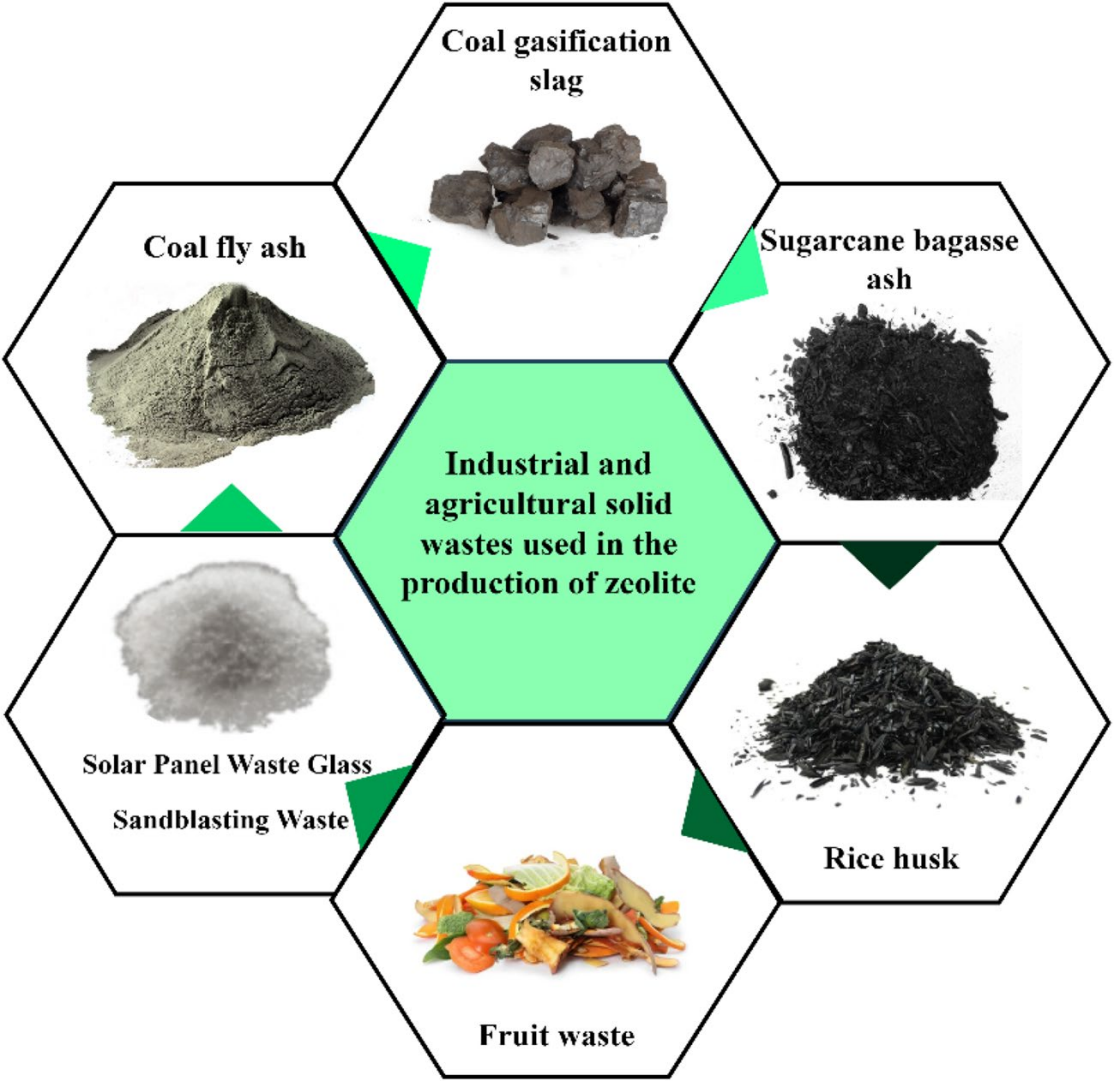
Industrial and agricultural solid wastes used in the production of zeolite

Although zeolites can be found as precipitates in nature, zeolites with higher purity and ion exchange capacity, as well as more uniform size and higher thermal resistance, can be synthesized from various high silica and alumina-based raw materials and thus have superior properties. The complex structures, unstable chemical compositions, and impurities of natural zeolites cause them to show lower performance compared to synthetic zeolites. This limitation in industrial applications has necessitated further study on synthetic zeolites. In addition to providing a significant reduction in process costs by using secondary resources such as waste materials in the process of obtaining synthetic zeolites, environmental problems are also prevented by contributing to the disposal processes of waste and turning them into value-added products. Waste materials containing Si and Al are becoming a source of interest in zeolite synthesis studies (Table 2). Figure 3 shows industrial and agricultural wastes that can be used as zeolite raw material sources.

**Table 2:** Advantages and disadvantages of various industrial wastes for zeolite synthesis (Zhang et al. 2022)

Advantages	Drawbacks
The synthesis of zeolite from waste is more cost-effective compared to conventional zeolites	CaO and carbon content in wastes can cause low zeolitization activity
The use of industrial wastes as sources of Al and/or Si leads to the valorization of by-products and waste recovery	
It is possible to produce pure and stable zeolites suitable for industrial applications

### Coal fly ash

The use of coal, which is the primary source of electricity generation, has shown a significant increasing trend over the years, and in this context, the increase in coal-based industrial waste has become remarkable. Coal fly ash (CFA) refers to fine mineral particles with an amorphous silica-aluminate structure, which are formed by burning coal in thermal power plants, accounting for approximately 70–90% of the amount of coal, and obtained from flue gases by means such as electrostatic and mechanical precipitation (Darmansyah et al. 2023; Patel et al. 2023). Transforming CFA into a value-added product is of great industrial interest as well as environmentally important, as it is a pollutant with toxic trace element content (Ahmaruzzaman 2010). This material, stored in waste collection sites, has become a health threat to living creatures living in the immediate environment.

**Fig. 4:**
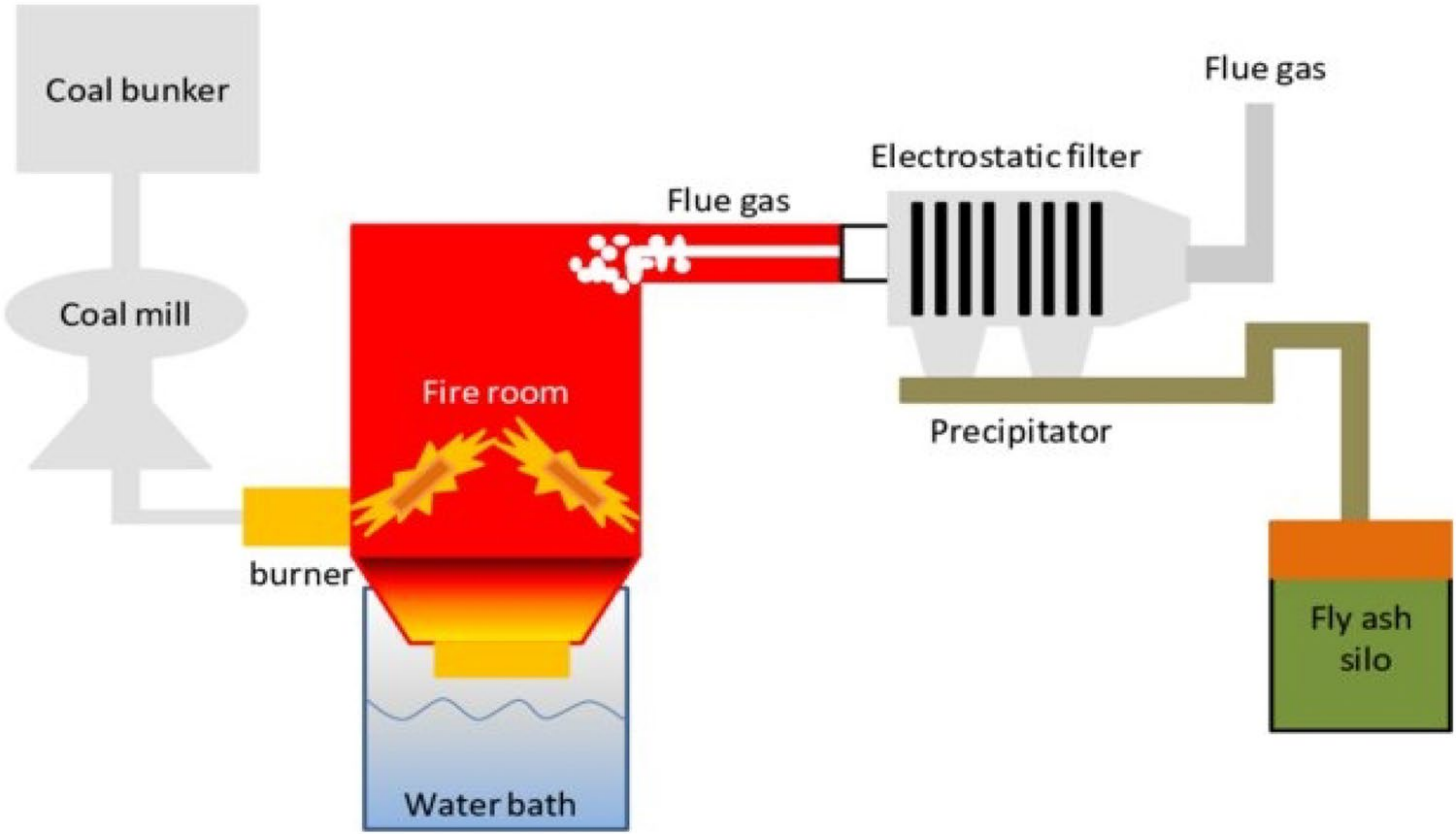
Coal fly ash production scheme (Technischen Fakultät & Toniolo 2019)

This material, which is generally gray and black in color, has an alkaline structure and has refractory properties, has a high specific surface area (0.2–10 m^2^/g), particle size between 1 and 100 μm, and a porosity of 30–65%. Its chemical composition is Si, Al, Ca, N, P, K, Mn, Fe, etc. It consists of elements and their oxides. CFA amorphous aluminosilicate and mullite (Al_2_O_3_·2SiO_2_) have crystal mineral content such as quartz, hematite (Fe_2_O_3_), and magnetite (Fe_3_O_4_) (Ndlovu et al. 2023). Approximately 0.15 t of fly ash per ton is formed during coal combustion (Iqbal et al. 2019) (Fig. 4).

### Coal gasification slag

**Fig. 5:**
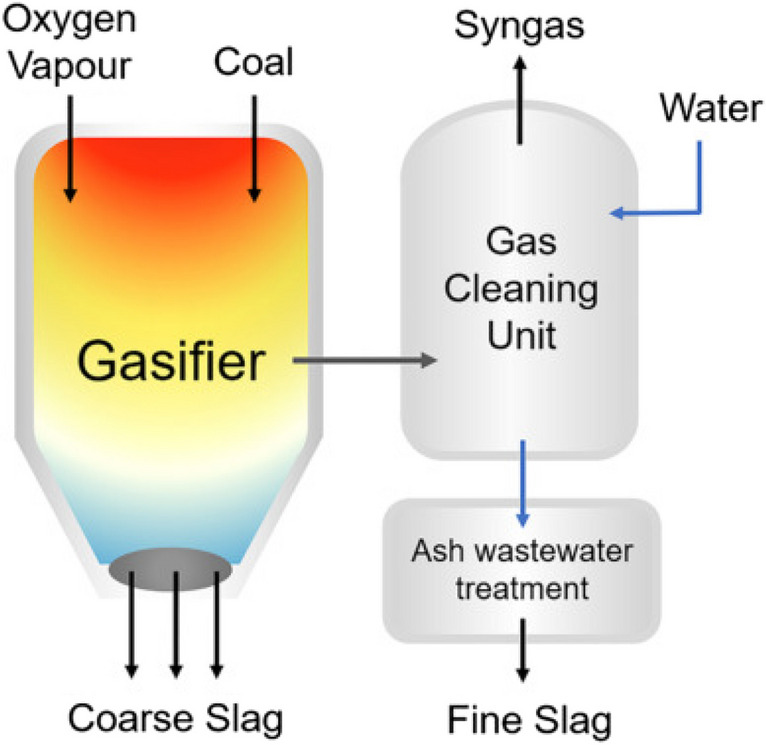
Schematic diagram of slag formation in coal gasification (Yuan et al. 2022a, b)

In the coal gasification process, a byproduct of fine slag (FS) from filters and coarse slag (CS) from lock chambers is formed (Yang et al. 2023). During the gasification process, coal particles decompose rapidly at high temperatures. Meanwhile, the minerals in the coal turn into slag. The molten part clinging to the gasifier wall and flowing to the bottom of the furnace cools and solidifies, turning into coarse slag. The small particles brought by the air flow are purified with synthesis gas and form fine slag (Cao et al. 2023). SiO_2_, Al_2_O_3_, CaO, Fe_2_O_3_, and residual carbon constitute the main components of gasification slag (Fig. 5). The concentrations of inorganic elements Si, Al, Fe, Ca, Mg, K, Na, and S in coal gasification slag are generally between 5 and 132 mg/g. In addition, it contains trace elements Zn, Zr, Co, Pb, Ni, Cu, As, Y, Ti, Ba, Sr, Mn, Cr, and Rb at values of 37–4063 μg/g. Particle sizes can be between 1.13 and 5.16 mm.

### Rice husk and their ash

Rice husks are hard protective coatings with a high silica content of 15–28% by weight, separated from the rice grains during the milling process in rice production. It is a by-product that occurs abundantly in rice-producing countries (Ng et al. 2015; Singh 2018). Rice husk ash (RHA) is obtained by burning rice husks. RHA appears grayish black in color due to unburned carbon in the structure. While amorphous silica is formed at combustion temperatures of 550–800 °C, crystalline silica is produced at higher temperatures. It generally consists of 85–98% silica by weight. Its purity depends on the combustion conditions for rice cultivation, the rice variety, and the climatic and geographical conditions of the region where it is grown. Although its specific gravity varies between 2.11 and 2.27, it is a highly porous and lightweight material and has a very high specific surface area (Ng et al. 2015; Siddique et al. 2020).

Rice husk is generally not recommended for use as animal feed due to its low nutritional value and difficult digestibility. Common disposal methods include open-field incineration and storage, but these methods cause energy waste, greenhouse gas emissions, air pollution, and land occupation (Ng et al. 2015). Contrary to all these problems, considering the high silica content of rice husk, various processes have been developed in which it is used as a raw material source in the production of zeolite, which is a value-added material, and studies have been carried out and are currently ongoing on the removal of unwanted materials with the resulting zeolite by the adsorption method (Chanda et al. 2023; Flores et al. 2021; Jin et al. 2023; Lee et al. 2023a, b; Tran-Nguyen et al. 2021).

### Solar panel waste glass and sandblasting waste

As the need for energy increases, coal, natural gas, and oil reserves used as raw materials are depleted and the use of these resources causes global warming. Among alternative energy sources, solar energy technology is a promising technology with its environmentally friendly and renewable features (Lee et al. 2023a, b; Sharma et al. 2021).

A steady increase in solar energy capacity is observed over time, but in this regard, the disposal of waste generated by the end of the panel life becomes an important issue. The lifespan of solar panels is between 25 and 30 years, and the accumulation of wastes at the end of this period in landfills causes environmental problems due to the fact that they contain toxic substances such as lead, while the loss of valuable materials requires the recycling of these wastes (Prasad et al. 2023). More than 80% of these silicon-based solar panels consist of glass material. Different technologies are being developed to reduce the amount of silicon used in solar cells. Thin-film solar cell technology and cutting technologies, which are among these technologies, have contributed to the decrease in the amount of silicon. However, sandblasting waste (SW) generated during the cutting process also requires a disposal process. SW is rich in alumina as well as silicon and silicon carbide content. The content of solar panel waste glass and sandblasting waste brought to mind the idea of using zeolite as a raw material source, since zeolites consist of silicates and aluminates, and it has been observed that studies have been carried out in this context (Lee et al. 2022, 2023b; Lin et al. 2022).

### Sugarcane bagasse ash

Sugarcane is the most produced crop worldwide. Large amounts of wet bagasse are released during the sugar production process, and the management of this waste is of great environmental importance. Applying the most common method, incineration, also creates another waste material, sugarcane bagasse ash (SCBA) (Payá et al. 2018).

Sugar beet bagasse ash (SCBA) is an industrial waste material of agricultural origin, the main component of which is silicon dioxide (Payá et al. 2017). Although its chemical composition varies depending on the source of SCBA, it consists of approximately 53–91% silica and 12–31% alumina element oxides. Due to its relatively high silica content, it is widely used in Portland cement blended mortars and concretes, aiming to improve the properties of concrete such as strength and durability (Pazouki et al. 2023; Soomro et al. 2023). In addition, there are studies in which SCBA is used as a raw material due to its SiO_2_ and Al_2_O_3_ content in order to obtain ion exchange materials at low costs. With techniques such as alkaline fusion, quartz particles in the structure can be dissolved and thus zeolites, which are silica-based materials, can be obtained (Dewajani et al. 2023; Hassan & Hameed 2023; Moisés et al. 2013; Oliveira et al. 2019).

### Fruit waste

Fruit production, which has a large place among biomass activities, is processed into different value-added products such as fruit juice and canned fruit, as well as for local consumption. However, all these processes, such as industrial production or final consumption, cause waste generation. These wastes consist of shells, seeds, and stems and are mostly accumulated in landfills. As it is known, the population continues to increase rapidly and in this context, due to the increasing need for food consumption, the amount of this type of waste is indirectly reaching gigantic sizes day by day (Plazzotta et al. 2017). This type of biomass waste is used in different sectors, and studies are continuing to use it in more sustainable areas. As a result of literature research, these wastes can be used for biogas fertilizers and energy production within the scope of energy recovery (ElMekawy et al. 2015; Shen et al. 2013), for the purpose of obtaining natural dyes in the textile industry (Bechtold et al. 2006), for composting purposes, etc. It has been observed that it is used directly to produce fertilizer with techniques (Chang et al. 2006) and as animal feed (San Martin et al. 2016) (Table 3).

**Table 3:** Chemical composition of industrial wastes

Solid waste type	SiO_2_	Al_2_O_3_	CaO	MgO	Fe_2_O_3_	K_2_O	Na_2_O	TiO_2_	LOI	Ref
Coal fly ash	53.02	22.74	5.86	1.82	5.75	2.07	0.10	0.94	6.4	(Moudar et al. 2022)
Coal gasification slag	53.44	17.21	10.12	1.92	11.23	2.27	1.33	-	12.55	(Ji et al. 2020)
Rice husk ash	68.28	1.28	0.37	3.59	0.54	3.14	3.78	0.07	13.33	(Abdullahi et al. 2017)
Solar panel waste glass	72.33	1.90	8.98	2.62	0.03	0.04	12.87	-	0.94	(Lin et al. 2015)
Sandblasting waste	10.25	45.60	5.47	-	12.23	0.16	1.23	21.10	-	(Lin et al. 2022)
Sugarcane bagasse ash	66.28	15.10	3.75	0.51	3.40	5.15	1.55	-	2.81	(Shahnaz & Shahzadi 2016)
Fruit waste	55.98	2.71	8.95	1.08	1.36	28.72	-	-	5.56	(Kilani et al. 2022)

Deionized, distilled, or industrial water is mostly used in zeolite synthesis studies. Few studies have analyzed the use of different water sources in zeolite synthesis, such as seawater (Belviso et al. 2009), aluminum corrosion byproduct (Hussar et al. 2011), industrial waste brine (Musyoka et al. 2011), and PEO process wastewater (Behin et al. 2014). Industrial wastewater was examined for zeolite synthesis and it was aimed to prevent high water consumption demand. Behin et al. (2014) studied the production of Na-A zeolite, a value-added product, by using CFA and PEO process wastewater. Plasma electrolytic oxidation (PEO) process is a technology that produces oxide coatings on the surfaces of metals and alloys such as aluminum, magnesium, and titanium. However, PEO process wastewater is considered an environmental problem and the disposal process requires high costs. With this study, two industrial wastes, CFA and PEO process wastewater, were processed and converted into products, thus eliminating environmental concerns in addition to capital savings. The zeolitized CFA they synthesized exhibited a high CEC value, leaching resistance against toxic elements, and good adsorption capacity. Based on the studies, it can be said that liquid wastes, in addition to industrial solid wastes, are promising candidates for zeolite synthesis. The potential to transform waste into value-added products is a promising field of study (Behin et al. 2014).

## Zeolite synthesis methods

Following the discovery of natural zeolites, the origin of zeolites synthesized by humans is based on the claim that levinite was prepared in the laboratory by St Claire Deville in 1862 (Cundy & Cox 2005). Zeolites synthesized as they are known today are based on the work of Richard Barrer and Robert Milton in the late 1940s. Barrer started his synthesis studies by using very high temperatures (170–270 °C) and strong salt solutions and obtained the first synthesized zeolite species (P and Q) (Barrer & Marcilly 1970). It was later determined that these synthesized materials had the KFI structure determined for zeolite ZK-5. Although Robert Milton used more reactive starting materials in his synthesis studies, he carried out the syntheses under milder conditions. As a result of his studies, he discovered zeolites A and X. In the following years, Milton and his colleagues synthesized 20 different types of zeolites, 14 of which were natural.

Following these studies in which only inorganic reaction components were used, the range of reactants was developed to include quaternary ammonium cations, and after the addition of organic compounds, the first high-content zeolite beta was discovered in 1967, and the archetypal high-silica zeolite type, ZSM-5, was discovered in 1972 (Cundy & Cox 2005).

In the following years, with the discovery of new materials and different techniques, understanding of reaction mechanisms, and development of characterization techniques, the discovery of new types of zeolite materials has continued until today, and studies are still being carried out with different techniques by focusing on the subject.

### Conventional hydrothermal synthesis method

In general, the hydrothermal synthesis method can be defined as the method of synthesizing crystals depending on the solubility of minerals in hot water under high pressure (Kafle 2020).

The hydrothermal synthesis method is the most popular method used in zeolite synthesis as an alternative to sol–gel and microemulsion methods, with its advantages such as low energy consumption, easy control of metastable phase formation, and high level-reactivity of reagents (Jin et al. 2021).

When examined in outline, the method involves mixing amorphous reactants containing silica and alumina with a cation source, mostly in a basic environment, and then heating the aqueous mixture in a closed stainless steel autoclave at temperatures exceeding 100 °C. As the temperature increases, the reactants remain in an amorphous structure and turn into a crystal zeolite product after the induction period. After sufficient time, the product completely turns into zeolite in the crystal phase. After this stage, the product is obtained through filtering, washing, and drying steps (Cundy & Cox 2005).

### Microwave-assisted hydrothermal methods

Bunmai et al. (2018) compared traditional (CH) and microwave-assisted hydrothermal synthesis (MH) methods in a study on the extraction of silica from cogon grass and its use in zeolite synthesis. Considering that the CH method follows a relatively slow process, they aimed to shorten this time with the microwave-assisted method. When the study results were examined, higher purity could be achieved due to homogeneous and rapid heating by using the MH method when synthesizing NaY zeolite. On the other hand, zeolites synthesized by the CH method were found to have higher crystallinity, particle size, BET surface area, and total acidity value. The NaY zeolite they synthesized by the MH method resulted in a larger outer surface and stronger acid strength (Bunmai et al. 2018).

Zhou et al. (2023), synthesized Na P1 zeolite from fly ashes formed by the combustion of domestic solid waste by microwave-assisted hydrothermal method, which is energy-saving, environmentally friendly, and cost-effective, without any pretreatment such as water washing, acid washing, or alkaline fusion. They have been carried out. Synthesis conditions were changed at 180 °C and for different times (0.5–2 h). The zeolite Na P1 they synthesized under optimum experimental conditions exhibited a large BET surface area of 61.42 m^2^/g and a high total pore volume of 0.44 m^3^/g (Zhou et al. 2023).

### Alkali fusion hydrothermal method

One of the most common methods used to convert materials that are sources of Si and Al, such as industrial or agricultural wastes, into zeolites is the hydrothermal method, in which they are mixed with an alkaline solution such as sodium hydroxide under different temperature, pressure, and reaction time conditions (Molina & Poole 2004).

Alkaline fusion hydrothermal synthesis method has been developed in order to find silica and aluminum sources in solid wastes, generally in inert minerals such as mullite and quartz, and to perform the extraction process effectively.

Ayele et al. (Ayele et al. 2016) compared traditional and alkaline fusion techniques in a study in which they synthesized zeolite A from low-grade kaolin. With the traditional hydrothermal synthesis method, we obtain rounded edge cubic zeolite A crystals with 75% optimum crystallinity and 250 mg CaCO_3_/g CEC, while with the alkaline fusion method, we obtain rounded edge cubic zeolite A with 84% optimum crystallinity and 300 mg CaCO_3_/g CEC value. Crystals were obtained. What is seen from the results of this study is that the alkali fusion method showed better performance in terms of time, energy cost, and even product quality compared to the traditional hydrothermal synthesis method (Ayele et al. 2016).

### Ultrasonic hydrothermal method

It is a method that involves the application of ultrasonic waves to the hydrothermal method. In zeolite synthesis processes, the ultrasonic-assisted hydrothermal method is a technique that has been preferred in recent years and has become popular over time, as it provides an increase in zeolite crystals and significantly shortens the synthesis time (Ng et al. 2019).

The application of ultrasonic-assisted hydrothermal synthesis provides a facile and versatile synthesis of nano- and microstructure compounds. There are chemical effects of ultrasound produced from acoustic cavitation through ultrasonic irradiation, which is a sonochemistry technology. Ultrasound is a high-frequency (20 kHz–10 MHz) sound wave compared to the upper limits of human hearing. When ultrasound is transmitted through a liquid–solid system, alternating expansive and compressive sound waves produce bubbles or voids when the pressure in the system drops below the vapor pressure. These cavities accumulate ultrasonic energy, and when they reach a certain size, they collapse, releasing the energy stored in the bubble in a very short time. The collapse of bubbles results in extremely high temperature, pressure, and cooling rate. This energy generated by the collapse of cavitation bubbles is sufficient for crystallization, and thanks to this energy, the crystal growth rate increases and results in an increase in the nucleation rate. Sonication-mediated hydrothermal process is an advantageous technique to shorten crystallization time (Cao et al. 2023; Ng et al. 2019).

It has been suggested that by using microwave and ultrasound energy sources, the reaction time can be reduced to minutes by causing homogeneous nucleation and uniform distribution of heat. An economically viable zeolitization process can be achieved by using alternative energy sources (Aldahri et al. 2016).

In all these methods, zeolites with different crystal structures are obtained by changing the reaction composition ratio, synthesis time, method heating temperature, and structural guiding chemical agents. Synthesis parameters of some studies that synthesized zeolite with different methods are given in Tables 4 and 5.

**Table 4:** Parameters of zeolite synthesis methods

	Parameters of conventional hydrothermal synthesis of zeolite									
Raw material	Alkali type			Conc. of alkali			Hydrothermal temperature	Hydrothermal time	Zeolite type	Ref
Waste glass and aluminum scraps	NaOH			2.5 M			60 °C	6 days	Na-P1	(Sayehi et al. 2022)
Coal gangue	NaOH			1.86 mol/L			95 °C	10.08 h	Na-A zeolit	(Jin et al. 2021)
	Alkali fusion-hydrothermal synthesis process parameters									
Raw material	Pretreatment		Alkali fusion part				Hydrothermal part		Zeolite type	Ref
	Calcine	Acid leaching	Alkali type	Ratio of Alkali/RM	Temperature	Time	Hydrothermal temperature	Hydrothermal time		
Jeju Island scoria	-	-	NaOH	0.6–2.4	530 °C	1 h	90 °C	5 h	Na-A zeolit	(Lee et al. 2018)
Coal fly ash	800 °C-1 h	-	NaOH	1:1.2	500 °C	1 h	80–100 °C	5–10 h	Zeolite X	(Sivalingam & Sen 2018)
Coal fly ash	700 °C-400 min	3wt%HCl 90 ℃ acid for 1 h	NaOH	1:1	650 °C	2 h	100 °C	24 h	Zeolite A	(He et al. 2020)
	Ultrasonic hydrothermal assisted zeolite synthesis process parameters									
Raw material	Alkali type		Conc. of alkali		Power of US	US time	Hydrothermal temperature	Hydro thermal time	Zeolite type	Ref
CHA	KOH or NaOH		-		200 W	2 to 4 h	353 K	6 to 96 h	Zeolite T	(Yin et al. 2023)
Sodium aluminate-collial silica	NaOH		-		-	30, 60, 120 min	100 °C	2 days	Zeolite RHO	(Ng et al. 2019)
Coal gangue	NaOH		0–5 mol/L		30 W	0 to 1 h	70–100 ℃	0.5–6 h	Hierarchical porous zeolites	(Zhou et al. 2020)
	Microwave-assisted hydrothermal synthesis of zeolite process parameters									
Raw material	Alkali type		Conc. of alkali		Power of MW	MW time	Hydrothermal temperature	Hydro thermal time	Zeolite type	Ref
Municipal solid waste incineration fly ash	NaOH		0–2 M		-	0.5 to 2 h	-	-	Na-P1	(Zhou et al. 2023)
SARs	NaOH				400 W	5 to 80 min	190 °C	0.5 to 6 h	SSZ-13	(Khan et al. 2023a, b)
SARs	KOH				800 W	0,5 to 24 h	160 °C	72 h	ZSM-22	(Muraza et al. 2015)
Kaolin	NaOH				800 W	6 min	-	-	Zeolite 4A	(Le et al. 2023)

**Table 5:** Advantages and drawbacks of zeolite synthesis procedures (Zhang et al. 2022)

Synthesis methods	Advantages	Drawbacks
Conventional hydrothermal synthesis	It is as simple as the process of synthesizing zeolite from pure materials	Hydrothermal crystallization requires a long reaction time. The yield and purity of the synthesized zeolite are lower
Alkali fusion-hydrothermal synthesis	It enables efficient activation of tailings and zeolites with high conversion and purity are obtained	The high temperature required for fusion increases the cost of the process
Ultrasonic hydrothermal synhtesis	It shortens the synthesis time of zeolite, improves the physicochemical properties of zeolite products, and can also reduce energy consumption	It can only promote the dissolution of the amorphous phase in solid waste rather than the crystalline phase. The mechanism of ultrasonically assisted crystallization is still not clearly understood
Microwave-assisted hydrothermal synthesis	The microwave heating method makes the heating speed and uniformity better than conventional heating. It effectively improves the solubility of tailings and zeolitization processes	Large-scale industrial application is lacking

## Application areas of zeolites

**Fig. 6:**
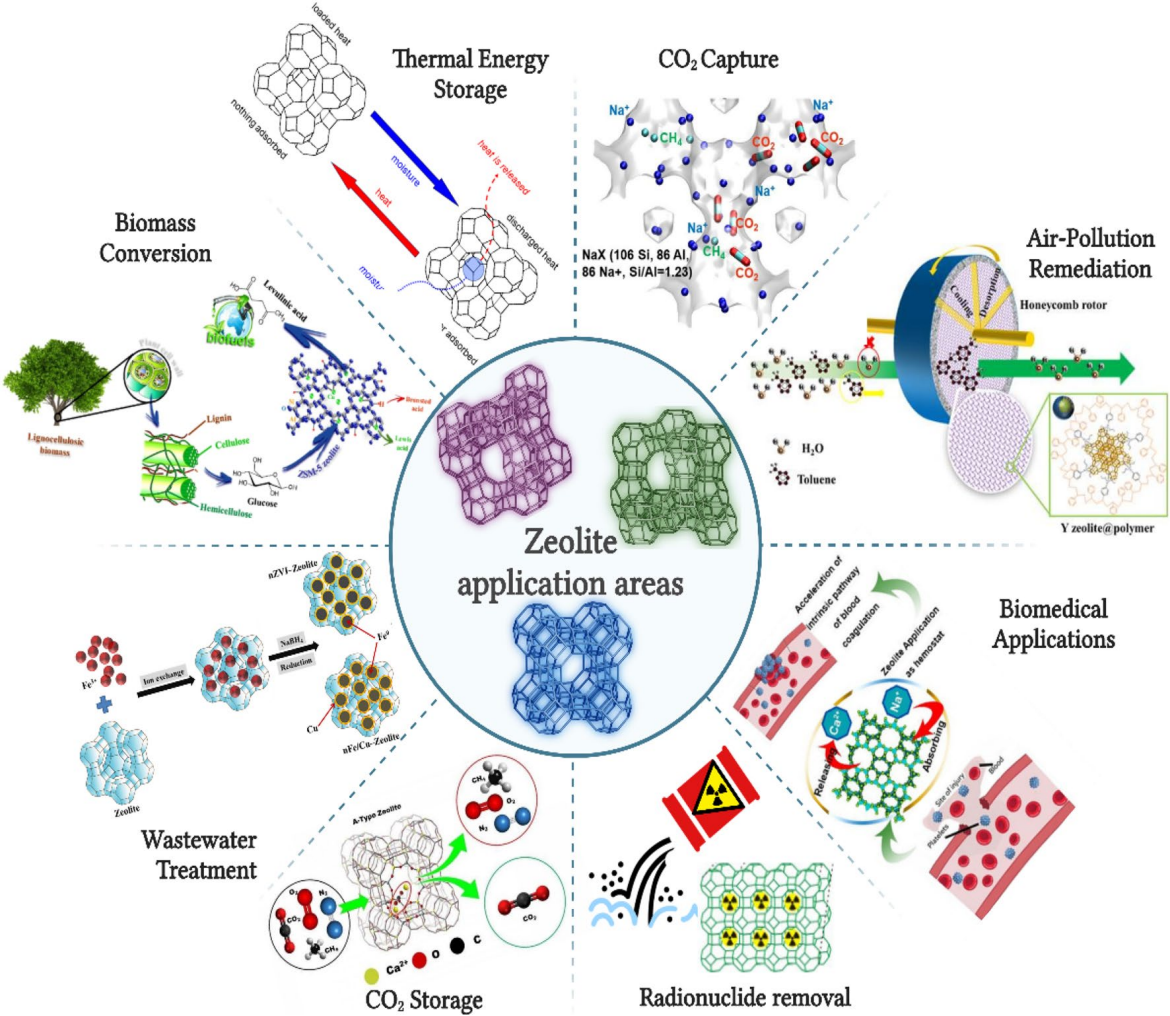
Zeolite application areas (Serati-Nouri et al. 2020; Shubair et al. 2019)

Zeolites commonly serve as catalysts in the conversion of biomass feedstocks into high-value fuels and chemicals. There are Brønsted acid sites, Lewis acid sites, and multifunctional active sites in the structure of zeolites, and thanks to these active sites, they provide a catalyst function in reactions (Dapsens et al. 2015).

Porous zeolites, also called molecular sieves, are used in the field of fuel cells to solve problems such as fuel transfer and membrane stability, to increase proton transport, to regulate fuel transitions, and to improve the flow of water in the electrode membrane. It serves as an electrode and catalyst in fuel cells and provides the transformation, reformation, and storage of fuel (Yeung & Han 2014).

It is important to develop systems that effectively use unused heat using adsorption–desorption cycles that can transport heat by recovering waste heat at low temperatures. Silica gel is generally used in closed systems to store thermal energy. Systems using zeolites have also been developed (Fig. 6). This method is a functional method that uses the drying and heat storage properties of zeolites (Fujii et al. 2022).

Carbon capture and storage are an effective method that reduces the release of CO_2_ into the atmosphere, but it is a very difficult process. Among different techniques such as cryogenic distillation and membrane separation, the adsorption method is one of the well-established methods that attracts much attention. Development of suitable adsorbents in adsorption technique is a current research topic. Among adsorbents, zeolites are promising for the CO_2_ capture technique with their homogeneous pore structures, highly specific surface areas, thermal and chemical stability, and qualitative properties (Rao et al. 2024; Tao et al. 2023).

Zeolties are used for the catalytic removal of NOx, NH_3_, and VOCs such as benzene, toluene, formaldehyde, and chloromethane, which generally originate from combustion engines and industrial and domestic products, in order to improve air pollution (Li et al. 2017).

It has wide applications in water treatment to solve the global water crisis by removing pollutants such as oil, heavy metals, radioactive waste, dyes, oils, and salts. The adsorption and ion exchange properties of zeolites provide selective removal of pollutants from wastewater, while reducing the risks of such pollutants on human health and the environment (Daer et al. 2015; Li et al. 2017).

Zeolites are also antibacterial, biocompatible, non-toxic, and highly absorbent, and with these features, they are preferred materials for use in the biomedical field. It is seen to be used in various biomedical applications such as drug delivery systems, wound healing, antibacterial and antimicrobial products, implant coating, removal of harmful ions from the body, hemodialysis, regenerative medicine, and tissue engineering (Serati-Nouri et al. 2020).

### Heavy metal

Heavy metals are elements with atomic weights between 63.5 and 200.6 and specific gravity over 5.0. These 23 metal elements include bismuth, tin, thallium, gold, arsenic, cerium, gallium, mercury, chromium, cobalt, antimony, copper, iron, cadmium, lead, manganese, nickel, silver, uranium, tellurium, platinum, vanadium, and zinc. These metals are classified as heavy metals (Velusamy et al. 2022).

Especially in rapidly developing countries, due to the increasing production amount in metal plating, mining, fertilizer, battery, and paper industries, more and more Sb, Cr, Cu, Pb, Zn, Co, Ni, etc. are being released into environmental waters. Heavy metals are mixed and the resulting wastewater is discharged directly or indirectly into the environment (Fu & Wang 2011). This direct discharge into sewage systems negatively affects biological wastewater treatment processes. It is known that exposure to these elements, which are not biodegradable and have the risk of accumulating in living organisms, poses a great risk for humans and other living things, even in trace amounts. Many heavy metal ions have toxic or carcinogenic effects, and the comparison of toxicity levels of heavy metals is as follows: “Co < Al < Cr < Pb < Ni < Zn < Cu < Cd < Hg” (Chipasa 2003). As heavy metals penetrate from the soil into the root systems of plants as a result of the mixing of surface waters, bioaccumulation in organisms and biomagnification in animals and foods occur (Velusamy et al. 2022). In humans, anemia occurs as a result of exposure to heavy metals, vomiting due to overdose of Zn, convulsions, and cramps as a result of Cu intake, and there are cases resulting in death. Chromium [Cr(VI)] exposure has the effect of causing tumor disease by causing disruption of DNA synthesis and mutagenic changes (Batubara et al. 2023).

Heavy metals also cause effects such as free radical release, cell damage, protein conformational change, enzyme inhibition, neuron damage, promotion of apoptosis, inhibition of neurotransmitters, and deoxyribonucleic acid (DNA) degradation (Mohod et al. 2013).

#### Heavy metal detection and treatment

Many different technologies have been implemented to determine the concentration and toxicity value of heavy metals (Asaithambi et al. 2020; Velusamy et al. 2021).

When the literature studies are examined, it is seen that studies have been carried out on the removal of heavy metals by various processes such as coagulation (Skotta et al. 2023; Zheng et al. 2021), chemical precipitation (Chen et al. 2018; González-Muñoz et al. 2006), ion exchange process (Dong et al. 2018; Zhao et al. 2019), membrane separation (Chen & Yang 2022; Zhao et al. 2023), reverse osmosis (Ozaki et al. 2002), and adsorption (El-habacha et al. 2023).

Among all these processes, the adsorption technique is a remarkable method with its advantages in heavy metal removal due to parameters such as low cost, easy and short-term applicability, and not requiring additional costs for sludge removal. (Anderson et al. 2022; Barakat 2011; Velarde et al. 2023). In the adsorption technique, finding an adsorbent that can be applied on an industrial scale is an important issue. In addition to examining technical properties such as adsorption capacity, selectivity, adsorption–desorption kinetics, and regeneration ability, the adsorbent is expected to have features such as long-term durability, low cost, and environmental friendliness (Velarde et al. 2023).

In the removal of heavy metals, activated carbon (Kong et al. 2022), carbon nanotubes (F. S. A. Khan et al. 2021), graphene oxides (Hosseinkhani et al. 2023), mesoporous silica, and carbon materials (Amin et al. 2023; Conte & Gómez 2024), clay particles (Ghasemi et al. 2023; Zhang et al. 2021), zeolites (Cheng et al. 2021a, b; Finish et al. 2023; Ren et al.  2022), and metal organic frameworks (Chu et al. 2023; Ragheb et al. 2022) are used. When all these methods are compared, it is a remarkable technology because zeolites can be obtained with low costs and energy requirements and can be used continuously (Velarde et al. 2023).

#### Heavy metal adsorption mechanisms of zeolite

**Fig. 7:**
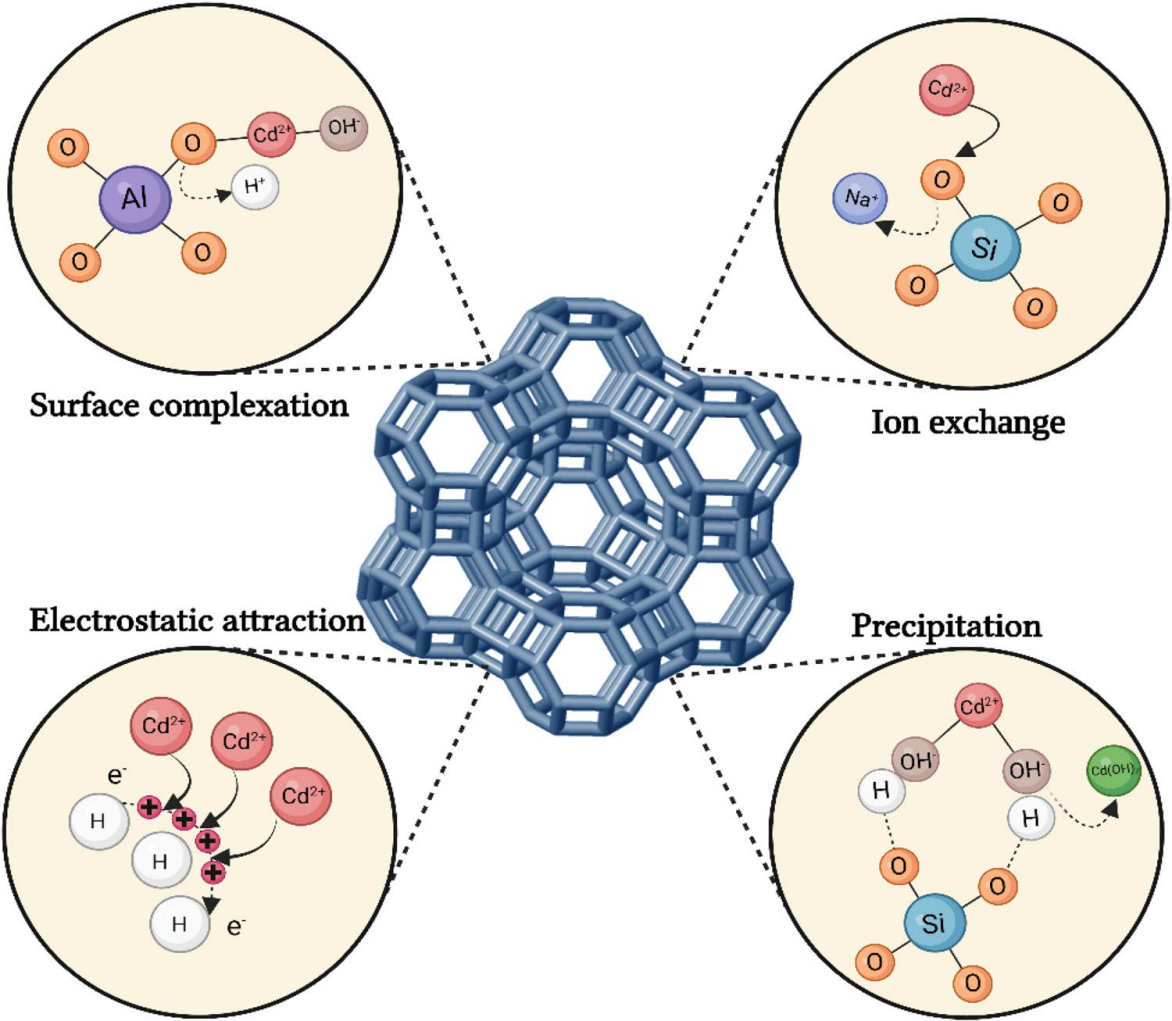
Adsorption mechanism of heavy metals on zeolites

The adsorption mechanism of heavy metal ions on zeolites has different potential processes. Predominantly heavy metal ions exist as exchangeable ions. In this case, ion exchange is one of the primary mechanisms for the adsorption of heavy metals. In addition, additional adsorption mechanisms such as precipitation, electrostatic attractions, and surface complexation may be effective. Aqueous oxides on the surface can form complexes by forming chemical bonds with metal ions (Fig. 7). Therefore, surface complexation occurs (Pal & Sen 2024).

### Dye

With the increase in industrial production in recent years, water pollution is one of the most common environmental problems. Wastes mixed into water due to the widespread use of dyes in different sectors such as the textile industry, food industry, dye industry, leather industry, cosmetics industry, plastic industry, and printing industry cause serious pollution (Pauletto et al. 2020). Mixing dyes into water prevents sunlight from penetrating into the water and prevents adequate oxygenation of the water, causing damage to the biological activities of microorganisms living in the water. Serious effects are also observed on human health, among which are allergies, dermatitis, respiratory toxicity, and it is known that it can even cause cancer (Pauletto et al. 2020; Somsiripan & Sangwichien 2023).

Dye molecules consist of chromophores, which have the ability to give color, and auxochromes, which have the ability to increase the color of the dye. The wavelength of the light absorbed by the chromophores and auxiliary chromiums in the structure determines the colors of the dyes (Khan et al. 2023a, b).

Dyes are generally divided into two groups: natural and synthetic dyes. Natural dyes are dyes that can be obtained from a variety of sources, from plant roots to insects and sea snail secretions. In the mid-nineteenth century, chemically produced synthetic dyes began to be used to ensure mass production and meet consumer expectations, especially in the textile industry. However, although synthetic dyes provide advanced coloring, they also cause water pollution (Mabuza et al. 2023).

**Fig. 8:**
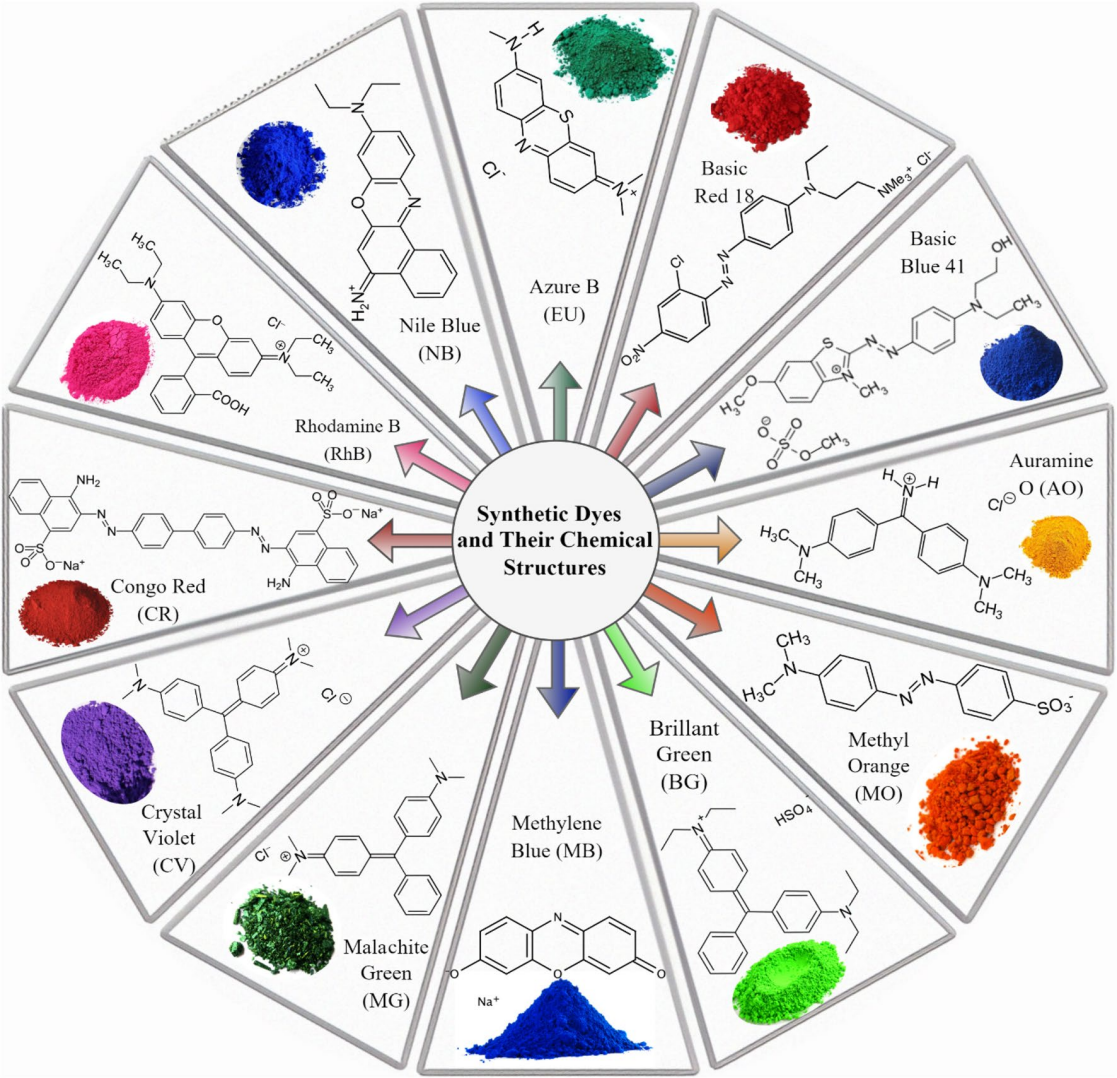
Synthetic dyes and their chemical structure

It has been stated that approximately 100 t/year of dye enters stream waters worldwide. However, even dyes in amounts less than 1 ppm in water have a significant effect. Different chemical, physical, and biological methods are used to remove dyes from water (Fig. 8). These methods include adsorption, coagulation, membrane separation, chemical oxidation, photocatalytic degradation, electrochemical, aerobic, and anaerobic microbial degradation techniques (Mu & Wang 2016).

#### Dye treatment

Many industrial production processes, especially the textile industry, produce dye wastewater. Discharging paint wastewater into surface waters poses a threat to the lives of humans, animals, and other living things, as well as environmental damage. Wastewater containing dyestuffs must be subjected to treatment processes before discharge in order to eliminate the risk of life-threatening environmental and other living things. Physical, physicochemical, and biological treatment technologies are used to treat this type of wastewater (Katheresan et al. 2018; Khan et al. 2023a, b).

It is known that traditionally used wastewater treatment technologies are ineffective in purifying synthetic textile dyes from wastewater due to the chemical stability of the dyestuffs (Forgacs et al. 2004).

Currently, there are numerous studies using various methods on dye removal in wastewater (Forgacs et al. 2004). Complete removal can only be achieved with treatment techniques that allow large-scale dye removal without producing secondary pollution. The most important feature expected in treatment methods is the disposal of dye without producing more hazardous by-products than wastewater (Rodríguez-Couto et al. 2009).

On the removal of dyes, adsorption (Wang et al. 2024), membrane filtration (Ma et al. 2022), ion exchange (Lahiri et al. 2022), and coagulation-flocculation are used (Hussein & Jasim 2021; Nnaji et al. 2022). Various physical and physicochemical treatment methods are used to purify wastewater by providing high removal efficiencies (Phi Long et al. 2024) and sonocatalytic (Phi Long et al. 2024) processes.

**Fig. 9:**
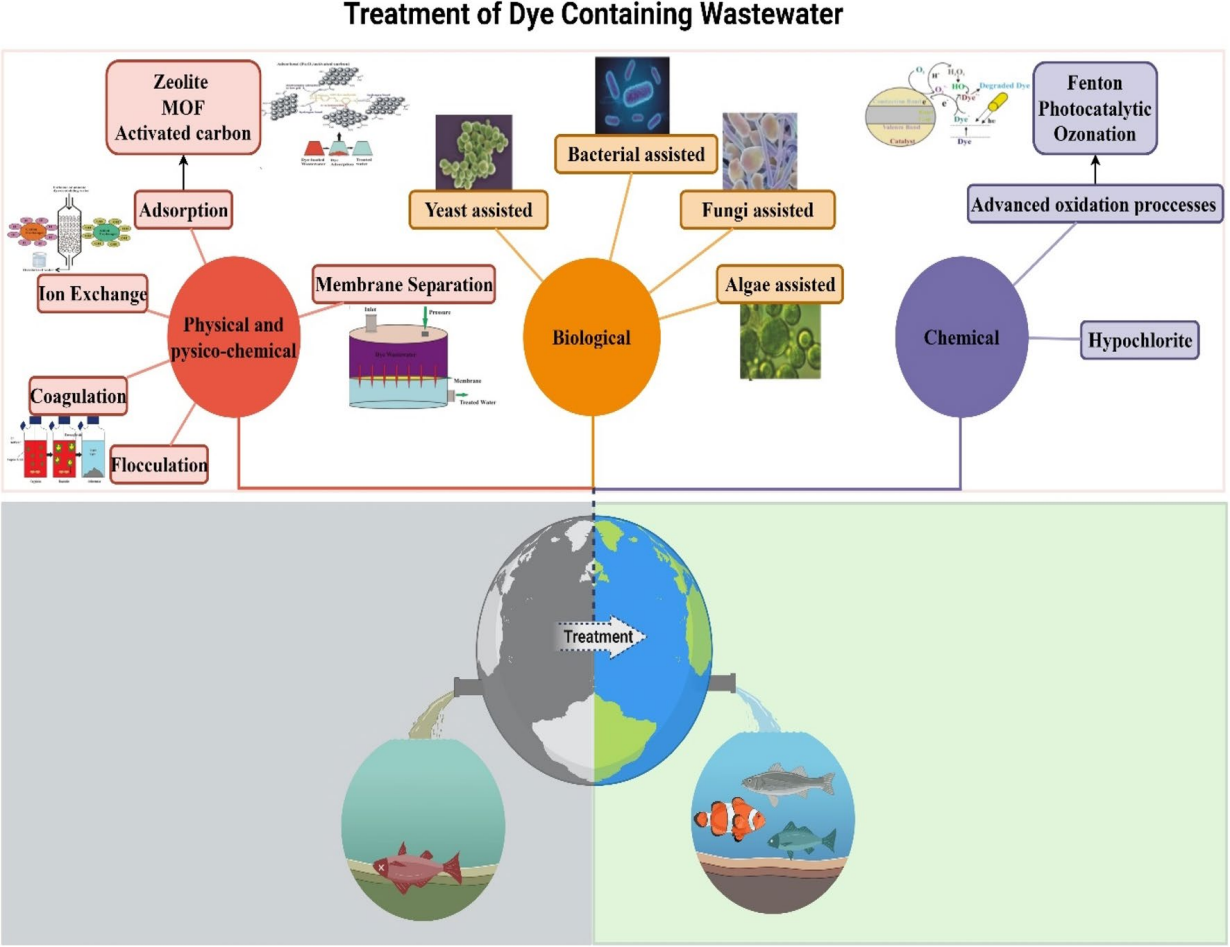
Treatment of dye-containing wastewater

In the bacterial purification process, which is one of the biological purification processes, the azo bonds of azo dyes, which are the most frequently used dye type especially in the textile industry, are reduced anaerobically, and the resulting aromatic amines are oxidized aerobically. With this method, firstly, the color removal of the wastewater is achieved, and secondly, it leads to a decrease in mineralization and toxicity values (Fig. 9). For this reason, the combined anaerobic–aerobic bacterial treatment process is a very attractive method for the treatment of wastewater containing dyes (Bonakdarpour et al. 2011).

#### Dye adsorption mechanism of zeolite

**Fig. 10:**
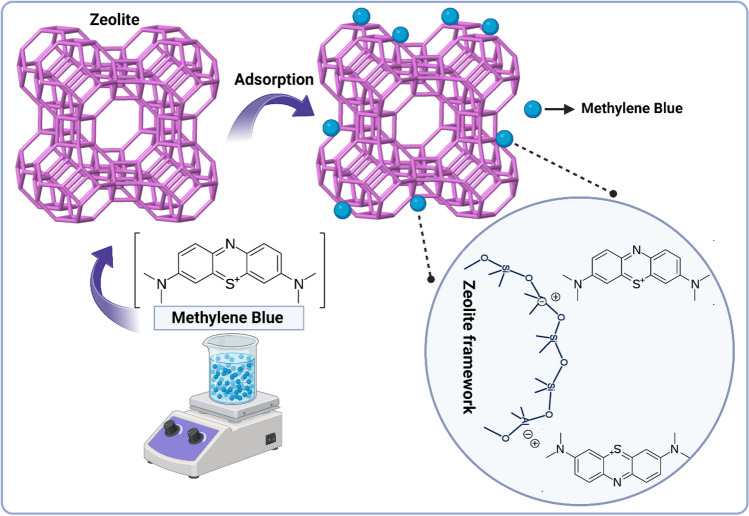
Dye adsorption mechanism of zeolite

As explained, zeolites are minerals with aluminosilicate structure, whose crystal structures consist of tetrahedral units in which [SiO_4_]^4−^ and [AlO_4_]^5−^ are bonded to each other through oxygen atoms. Thanks to the isomorphic substitution ability of Si^4+^ in the structure, which can replace Al^3+^, zeolites produce negative highs that can easily bond with other cations. These negative charges, which are permanent and do not depend on the pH condition in the structure, are capable of being replaced by positively charged ions. In this way, cationic dyes that carry a positive charge, such as methylene blue, can be adsorbed on this surface (Fig. 10). The adsorption ability of zeolite depends on the density of negative charges on the surface (Prajaputra et al. 2019).

Studies on the removal of heavy metals and dyes from wastewater with zeolites synthesized with different techniques are compiled in Table 6, which includes adsorption capacities, surface areas, and pore volumes.

**Table 6.**
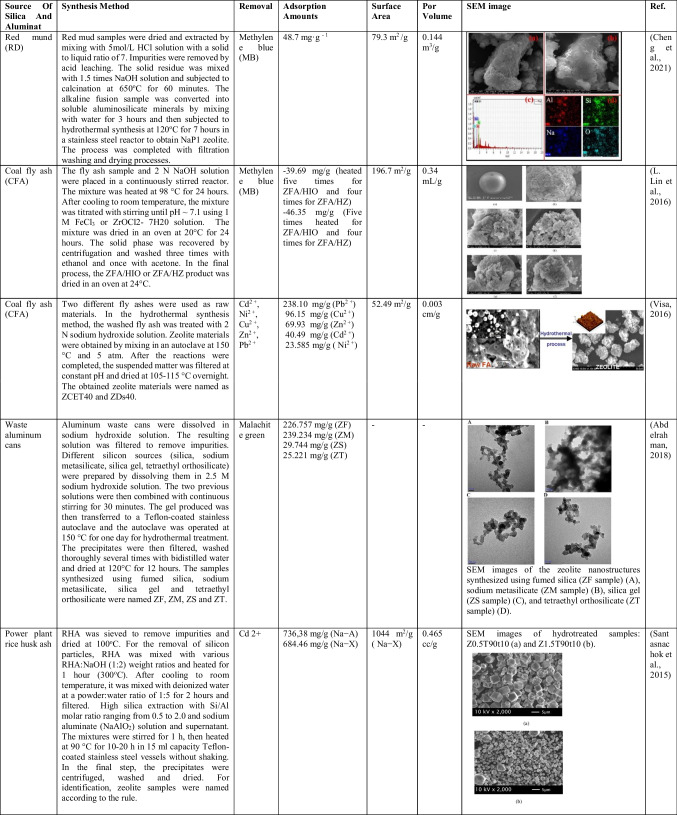
Studies on the removal of heavy metals and dyes from wastewater with zeolites synthesized by different techniques

## Characterization of the material

### Importance of characterization

Characterization tests are carried out for the development and commercialization of new zeolites, zeolytic catalysts, and adsorbents. Since no single method is sufficient, a combination of selective characterization techniques must be performed. In this section, various characterization techniques that are most frequently used are included.

### X-ray diffraction (XRD) analysis

X-ray powder diffraction is a central characterization technique used to determine the structures of zeolites. With this technique, the synthesis of a new zeolite is proven, and conclusions are reached about whether the synthesized zeolite has the desired properties and how much crystallization has occurred. In addition, the powder diffraction method is probably the most widely used method in identifying impurities in the synthesized zeolite, understanding the effect of experimental parameters such as heat treatment, and explaining the extent to which zeolite is bound to the catalyst or adsorbent pellets (Bradley et al. 2010).

**Fig. 11:**
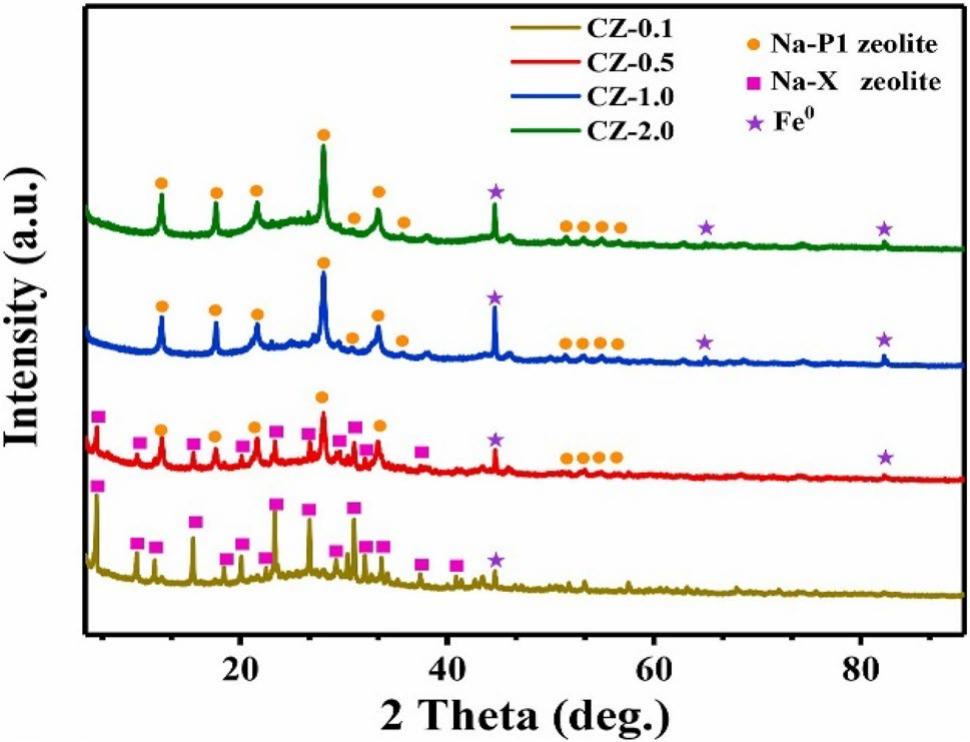
XRD graph of Na-P1 zeolite synthesized from coal gasification fine slag (Shu et al. 2023)

XRD analysis is used for phase identification. Zeolite type identification can be made by taking advantage of the fact that zeolites of the same structure type show similar powder patterns. However, if there is more than one phase in the structure, the powder pattern is a superposition of the separate phases and the overall density at the peaks is related to the amount of each phase. For these reasons, the identity and quantity of each phase can be determined with the powder patterns obtained from phase mixtures. The ratio of the peaks in the powder diffraction patterns of zeolite samples to the same peak intensity as the reference zeolite is usually determined as a percentage, and this expression is defined as the percentage of zeolite crystallinity. In other words, this definition represents the amount of crystalline zeolite in the sample zeolite compared to the reference zeolite (Fig. 11). SiO_2_ and Al_2_O_3_ polymorphism of untreated raw material is determined by XRD analysis. In addition, zeolite structures are clarified by comparing the XRD patterns of zeolites synthesized depending on different SiO_2_/Al_2_O_3_ molar ratios and by comparing the main peaks in the XRD patterns with the standard peaks of the zeolites (Tran-Nguyen et al. 2021).

Shu et al. (2023) compared the XRD models of samples to which they applied acid leaching at different concentrations of 0.1, 0.5, 1.0, and 2.0 mol/L HCl in their study in which they synthesized Na-P1 zeolite from coal gasification fine slag. They observed that A-type zeolite was formed at a Si/Al ratio of 0.94–1.05, X-type zeolite was formed at a ratio of 1–3, and Na-P1 zeolite was formed at a higher Si/Al ratio. In the zeolite sample (CZ-1.0) studied at 1.0 mol/L HCl, the Si/Al ratio increased to 5.7, exceeding the critical point defined for zeolite X, and it was understood that relatively pure Na-P1 zeolite was formed. In the CZ-2.0 sample, where the HCl concentration was increased, it showed the same diffraction peaks, although a decrease in the impurity level was observed. Based on the XRD results, they determined the acid concentration required for the synthesis of pure Na-P1 zeolite (Shu et al. 2023).

### Electron microscopy characterization of zeolitic systems

Electron microscopy technique is frequently used to understand the structural, morphological, and compositional properties of zeolite materials. There are three basic application methods of electron beam welding. Diffraction and high-resolution TEM imaging determine bulk and defect structures, surface morphology is determined by TEM and SEM methods, and compositional zeolite characterization is determined by EDS (energy dispersive X-ray spectroscopy) method. These imaging methods, which have been used in zeolite materials dating back to the 1950s, were generally aimed at structural and morphological characterization, and with the commercial use of SEM and EDX techniques in the 1960s, the determination of surface morphology and chemical compositions became possible. With the introduction of the TEM imaging technique in the 1980s, it became possible to directly examine the details of zeolite structures. One of the most technologically important features of zeolites is their acidity, provided by hydroxyl groups due to substituent cations in their framework structures. However, imaging studies performed with electron microscopes are limited in detecting acidity in the structure and characterizing the zeolite structure due to reasons such as weak scattering of the transmitted electron by a hydroxyl group and small atomic number differences of substituted cations. Fortunately, the acidity of zeolites can be determined by different methods such as NMR and FTIR (Shu et al. 2023).

### Scanning electron microscopy (SEM) analysis

Surface morphology studies of zeolite materials can be carried out comprehensively using the SEM technique. In addition to the stated advantages of this imaging technique, which does not require special preparation and is applicable to grain morphologies larger than a few microns, it is also generally easy to interpret.

Most zeolite applications require the introduction of a feed or absorbent into the zeolitic micropores. Assuming that the crystal diameter is *d*, in most cases, the rate of occurrence of this phenomenon depends on 1/*d*2 in the 3D pore system and tends to increase with the density of pore openings. For these reasons, crystal morphology is a dominant feature that shows the performance of the material in zeolite applications such as catalysis and adsorption.

**Fig. 12:**
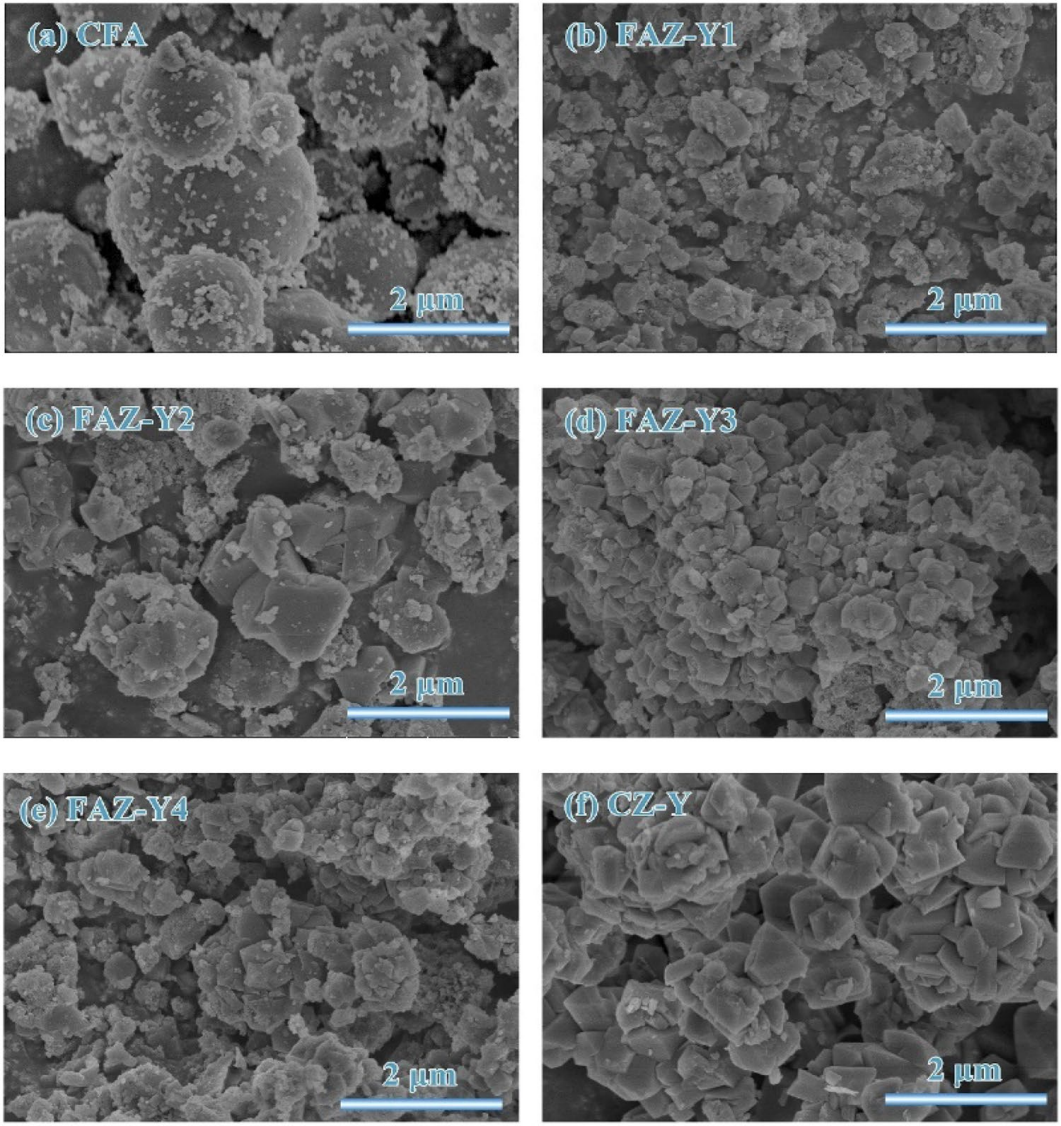
SEM images of commercial zeolite Y synthesized using coal fly ash (CFA) (Ren et al. 2020)

Ren et al. (2020) synthesized commercial zeolite Y using raw coal fly ash (CFA) and evaluated the zeolite products they synthesized under different conditions with SEM micrographs. They understood that the raw material CFA has a spherical shape (Fig. 12a), the surface changes during the zeolitization process, and the irregular shapes formed are zeolite structures. While defective octahedral particles with a rough surface and a wide range of sizes between 0.29 and 1.2 μm are observed in Fig. 12c, its structure with irregular morphology was observed to transform into uniform submicron octahedral particles with narrow particle sizes and highly crystallized particles in Fig. 12d. While the morphologies of Fig. 12d and f are the same, octahedral particles and some defective particles are observed together in Fig. 12e. They stated that this may be due to the re-dissolution of the crystal as the crystallization time increases. In the study, SEM and XRD results were evaluated together and consistent results were obtained (Ren et al. 2020).

Take it again (2023), in their study where they synthesized zeolite T by ultrasonic pretreatment of CHA and then hydrothermal synthesis method, the effects of using NaOH and KOH solutions in the pretreatment were evaluated by analysis. Combining the XRD results and SEM results of the products subjected to KOH solution, it was concluded that the ultrasonic treatment applied at 313 K was better than higher temperatures and that the appropriate ultrasonic condition could not only reduce the size of the crystals but also increase their purity. In addition, they discovered that KOH and NaOH direct the structure during the interzeolite transformation process. The final products were greatly affected by additional alkali metal ions (Yin et al. 2023).

### FT-IR analysis

One of the most commonly used methods in the examination of zeolites is FTIR (Fourier transform infrared) spectroscopy. It is a very successful technique in identifying the mineral structures, bonds, and chemical properties of zeolites (Volkov et al. 2021). IR spectra are used to express the acidity and concentrations of functional groups attached to the structural chains of zeolites. In addition, the catalytic properties of zeolite samples can be predicted (Li 2005).

Mid-infrared spectra (4000–400 cm^–1^) were taken into account in the characterization of zeolites by FTIR. In the interpretation of infrared spectra, specific zeolite structure type, group, secondary structure units such as double rings and large pore openings are evaluated. The main structural groups of the zeolites synthesized in the studies are determined from infrared patterns. Frame structures of zeolites can be expressed as inside fingers. Infrared spectra in the 1500–400 cm^−1^ region are indicative of qualitative characterization.

Infrared spectra of zeolites in the 1300–400 cm^−1^ region generally consist of two vibration classes: (i) originating from the internal vibration of the TO_4_ tetrahedron framework, which are the primary structural units in zeolite frameworks, and (ii) vibrations arising from the external connection between tetrahedral units sensitive to the framework structure and the presence of secondary structural units, the building blocks of polyhedral such as rings and pores. Characteristic FTIR bands of zeolites are given in Table 7 (Suresh Kumar 2007).

**Table 7:** Characteristic FTIR bands of zeolites (Suresh Kumar 2007)

Internal tetrahedral linkages	Assignment (cm^−1^)
Asymmetrıc stretch Asymmetric stretch vibrations of bridge ties—νas Si–O(Si) ve νas Si–O(Al)	1250–950
Symmetric stretch Symmetrical stretch vibrations of bridge ties—νs Si–O-Si Symmetrical stretch vibrations of bridge ties—νs Si–O-Al	720–650
T-O bend Bridging OH groups in Al–OH-Si	5000–420
External linkages	Assignment (cm^−1^)
Double ring	650–500
Pore opening	420–300
Symmetric stretch	820–750
Asymmetrıc stretch	1150–1050 shoulder
(Complex band) symmetric stretching vibrations νs of bridging bonds Si–O-Si and bending vibrations—δ O-Si–O	555 cm^−1^
Bending vibrations—δ O-Si–O occurring “antiphase”	468 cm^−1^
Bending vibrations—δ O-Si–O ve δ O–Al-O	377 cm^−1^

A characteristic sharp peak is observed in the FTIR spectra of zeolites around 1100 cm^−1^. This peak is unique to hydrated three-dimensional networked zeolite structures, and this vibration is primarily associated with oxygen atoms and is attributed to a T–O stretching involving ← O, T →  ← O movements. The position of Si–O varies depending on the number of electronegative groups in the zeolite. Therefore, the Si–O frequency is sensitive to displacement. When there is an OH or NH group nearby, the stretching frequency due to hydrogen bonding in the structure decreases to 500–800 cm^−1^. The broad band around 1100 cm^−1^ is attributed to the asymmetric stretching of SiO_4_ tetrahedra. The transition to a relatively high wave number is caused by the presence of large amounts of different cations due to the Si–O bond distance being shorter than the Al-O bond distance. The presence of a weaker shoulder at 1000 cm^−1^ is attributed to the vibration involving ≡Al–OH gaps formed by cation gaps. Many different frequencies reflecting the lattice composition are observed in the region of 750 cm^−1^ and below. The absorption peaks at 750–700 cm^−1^ correspond to the symmetric stretching vibrations of SiO_4_ groups. The bands around 649, 544, and 468 cm^−1^ indicate bending vibration of SiO_4_ groups or vibration of four-membered silicate chain rings.

Symmetric stretching modes in the lower spectral region of 720–650 cm^−1^ shift to the higher region of 820–750 cm^−1^ with the inclusion of a tetrahedral stretch. The weak absorption bands at 650 cm^−1^ are due to the interaction between the alumina tetrahedra and Al^3+^ in the zeolite structure, and the splitting state of the peak in this region indicates that there is high O–Si–O in the structure (Suresh Kumar 2007; Tabassum et al. 2018).

With lattice vibration intensities around 558 cm^−1^, small nuclei concentrations of atoms in the molecule occurring during the induction period can be monitored. The increase in this vibration represents the increase in the double ring numbers of T atoms. The absorptions here arise either from TO_4_ bending or from movements of the outer link of SiO_4_ and AlO_4_ tetrahedrals. Weak bands can be observed around 1480–1380 cm^−1^ due to the effect of excess alumina in the pores. It is possible to see the effects of water molecules and amines on the stretching vibration of the hydrogen group with wide weak bands around 4000–3000 cm^−1^. The OH stretching modes at wave numbers with the shoulder at 3600 cm^−1^ and 3626 cm^−1^ originate from two different Brönsted zones as well as 3678 cm^−1^ (Si–OH) and 3743 cm^−1^ (Al–OH). While the bands at 3660 cm^−1^ correspond to OH groups associated with extra Al species in the cage, it is stated that the band at 3690 cm^−^1 corresponds to the Al–OH type, and the OH band around 3740 cm^−1^ may result from blocked OH groups on the zeolite surface. The OH band around 3650 cm^−1^ originates from non-acidic OH groups attached to [AlO]^+^ species in most cases, and the OH band aroundlite − 1 types have similar spectral properties, such as the internal tetrahedral mode, which is less sensitive to change in lattice structures, some structural differences can still be observed (Fan et al. 2012; Suresh Kumar 2007).

### Solid state 29Si and 27Al magic angle spinning (MAS) nuclear magnetic resonance (NMR)

NMR technique, one of the spectroscopic analysis methods, is a powerful technique in the characterization of zeolite materials themselves and the adsorbate types involved in the chemical reaction in their applications for catalyst purposes.

With the MAS NMR technique, NMR spectra of solid samples have become comparable to liquids in terms of spectral resolution. It is an effective technique in elucidating the structural properties of solid materials such as zeolite. Aluminasilicate framework characterization of various zeolites is performed using the 29Si and 27Al MAS NMR technique. With this technique, the surface hydroxyl groups of zeolites, Brønstead acidity, porosity, and adsorption sites with adsorbed probe molecules can be identified (Stepanov 2016). Solid nuclear magnetic technique is an effective method used to analyze the structure of zeolite frameworks. Although zeolites are highly crystalline materials, the small size of the crystals (usually on the order of a few microns) precludes the direct application of single crystal diffraction techniques. Solid-state NMR spectroscopy interrogates local geometry and short-range arrangements, corresponding to much longer-range periodicities. In zeolites with low Si/Al ratio, five resonances are observed, which describe the average Si and Al distributions throughout the framework: Si-[4A1], Si[3Al,1Si], …. Si[4Si] (Jacobs & Santen 1989). In zeolite materials with high Si content, 29Si spectra are directly related to the lattice structure. With the removal of Al, it can be seen that all silicones form a perfectly ordered system in the same local framework (Si-[4Si]). In this case, 29Si resonances are observed; quite narrowy signals are observed for structures with crystallographically non-equivalent regions that directly reflect the numbers and occupancy of asymmetric T regions in the unit cell. While the technique is known to be more sensitive to local arrangements than diffraction data, exact structure determination and the effects of temperature and interaction on organic structures are clarified more clearly by using it together with syncrotron-based powder X-ray diffraction measurements (Fyfe et al. 1990).

**Fig. 13:**
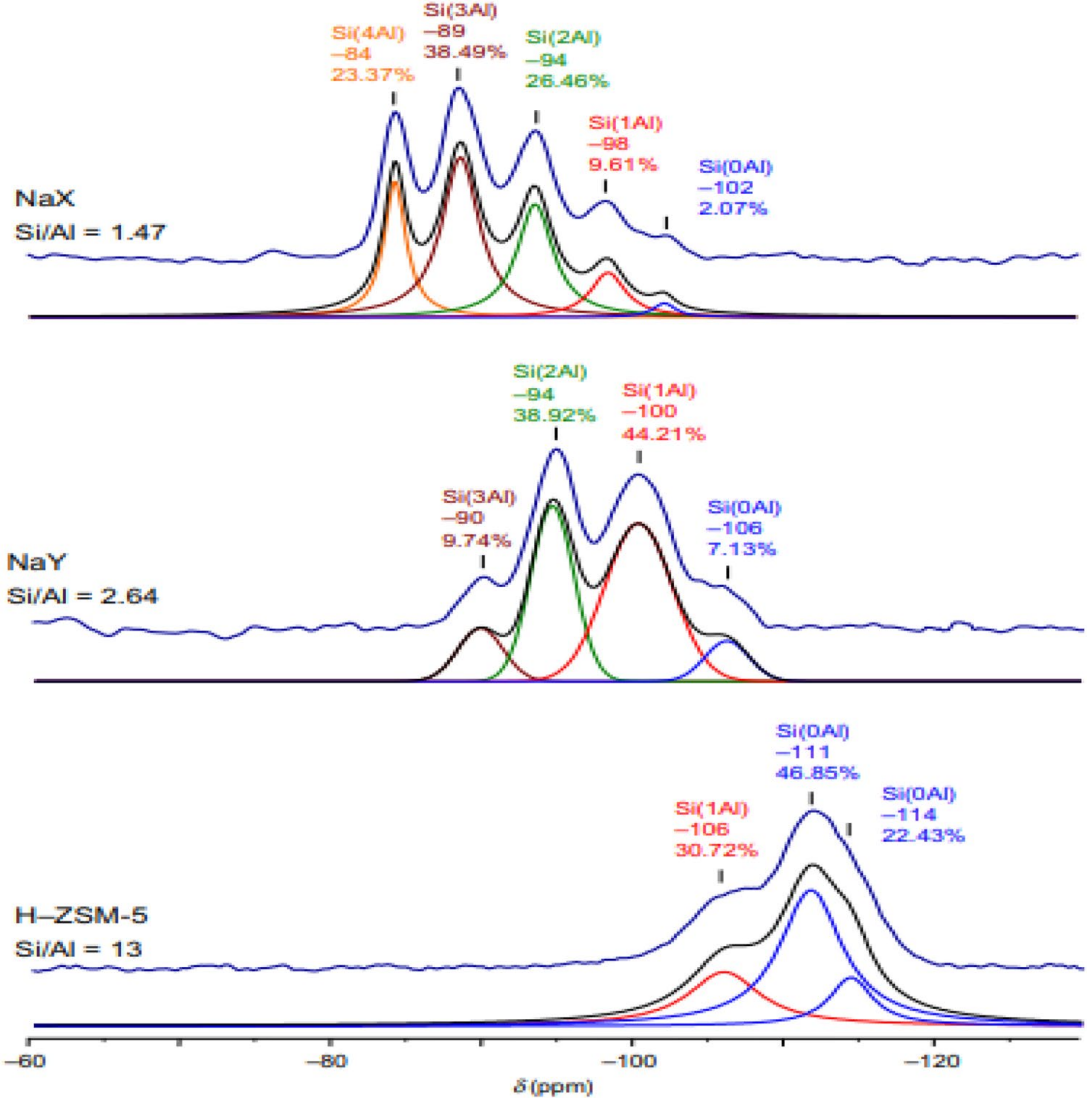
29Si MAS NNR spectra of some zeolites: NaX (Si/Al = 1.47), NaY (Si/Al = 2.64), H–ZSM-5 (Si/Al = 13) (Kneller et al. 2003)

The signal resulting from the tetrahedral aluminum in the zeolite structure can be distorted such that a significant amount of signal is lost when the zeolite is dry, which is related to the immobility of exchangeable cations in the structure and the oxygens bound to the aluminum. In the aluminum region, the resonance broadens with the transition from tetrahedral symmetry to trigonal–bipyramidal symmetry (Fig. 13). This is resolved by hydrating the samples before obtaining the 27AlNMR spectrum (Kneller et al. 2003).

### Electronprobe micro-analyzer (EPMA)

EPMA is a technique used for micrometer-scale quantitative analysis of the elemental compositions of solid samples. By keV electron bombardment sent onto the sample, characteristic X-rays are excited and characterized by wavelength dispersive (WD) spectrometers. In addition, detailed mapping of the compositional contrast of the structure is provided with the quantitative analytical imaging technique (Llovet 2019). It is seen that the EPMA technique is used in different fields, especially in geology, especially for mapping the spatial distribution of large and small elements in solid samples (Trueman et al. 2019). EPMA) technique is preferred in order to better understand the atomic substitutions of zeolite mineral compositions and to make geological and geochemical interpretations (Campbell et al. 2016). With the EPMA WDS mapping technique, the average element mass distributions on the surface of element-dispersed zeolite particles are determined. While WDS mapping spectra show the average mass content of elements (Si, Al, Na) completely quantitatively, the %mass values of the elements are reached (Chen et al. 2023).

**Fig. 14:**
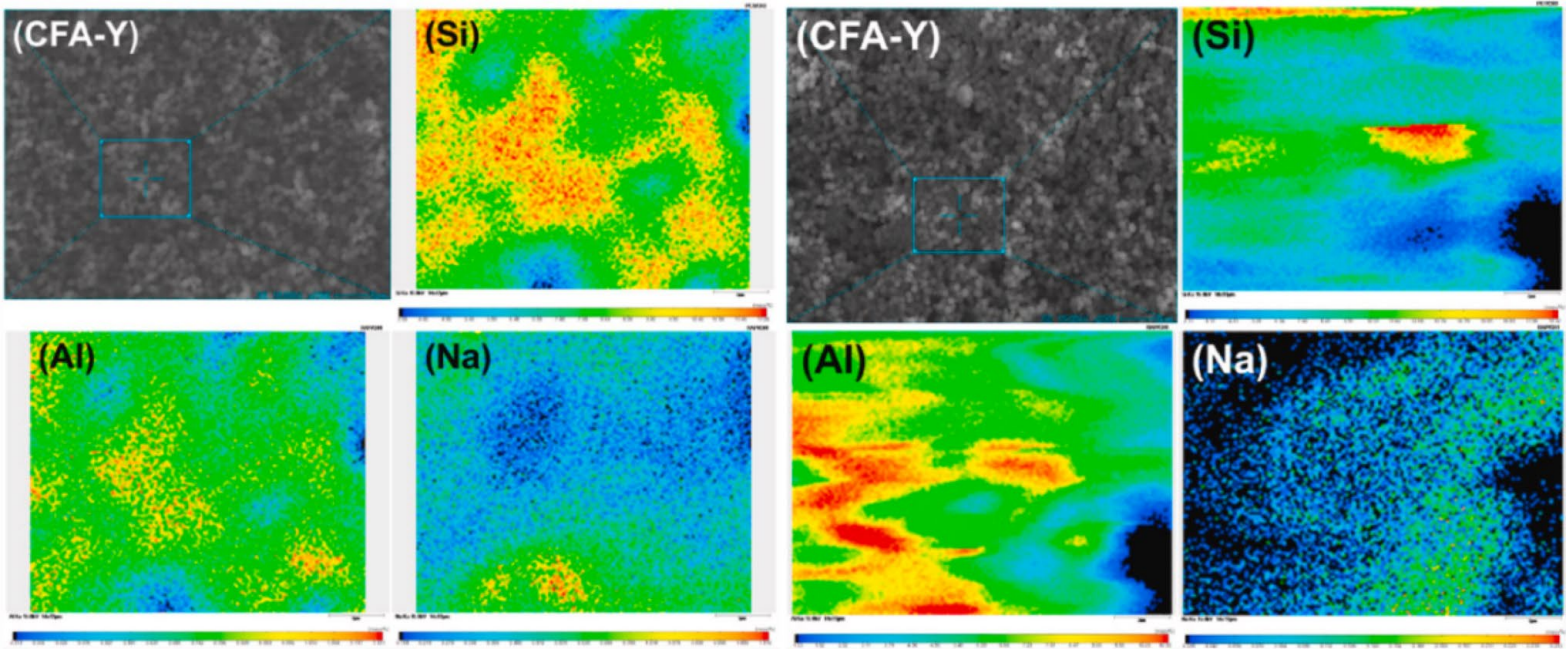
EPMA WDS elemental (Si, Al, Na) mapping spectra of CFA-Y and H-CFA-Y (Z. Chen et al. 2023)

As a result of EPMA WDS mapping of the zeolites synthesized in the studies, information is obtained about the homogeneity of element distribution and the average mass contents of the elements (Si, Al, Na) are determined. It offers the opportunity to compare the effects of zeolites synthesized under different conditions or with different raw material ratios on the surface framework with mass% values (Campbell et al. 2016) (Fig. 14).

Chen et al. (2023) synthesized pure Y (FAU) zeolite from coal fly ash (CFA) using the combined method assisted by microwave and ultrasonic irradiation. In order to precisely measure the element concentration on the surface of zeolite samples and to determine the weight % and mole percentage of the elements (Na, Al, and Si), quantitative analysis was carried out at three randomly selected positions with the EPMA technique. Si/Al mol% and Si/Na mol% ratios of CFA-Y and H-CFA-Y synthesized at different temperatures and times were found. In addition to the CFA-Y synthesis steps, H-CFA-Y zeolite was subjected to ion exchange in NH_4_NO_3_ (1.0 M) solution at 60 °C for 3 h. In the EPMA results, it was understood that NH_4_^+^ ion exchange and NH_4_^+^ calcination led to a significant decrease in the Si/Al mass % ratio on the surface framework of the H-CFA-Y zeolite and caused a significant removal of Na^+^ (Chen et al. 2023).

### Atomic force microscopy (AFM)

**Fig. 15:**
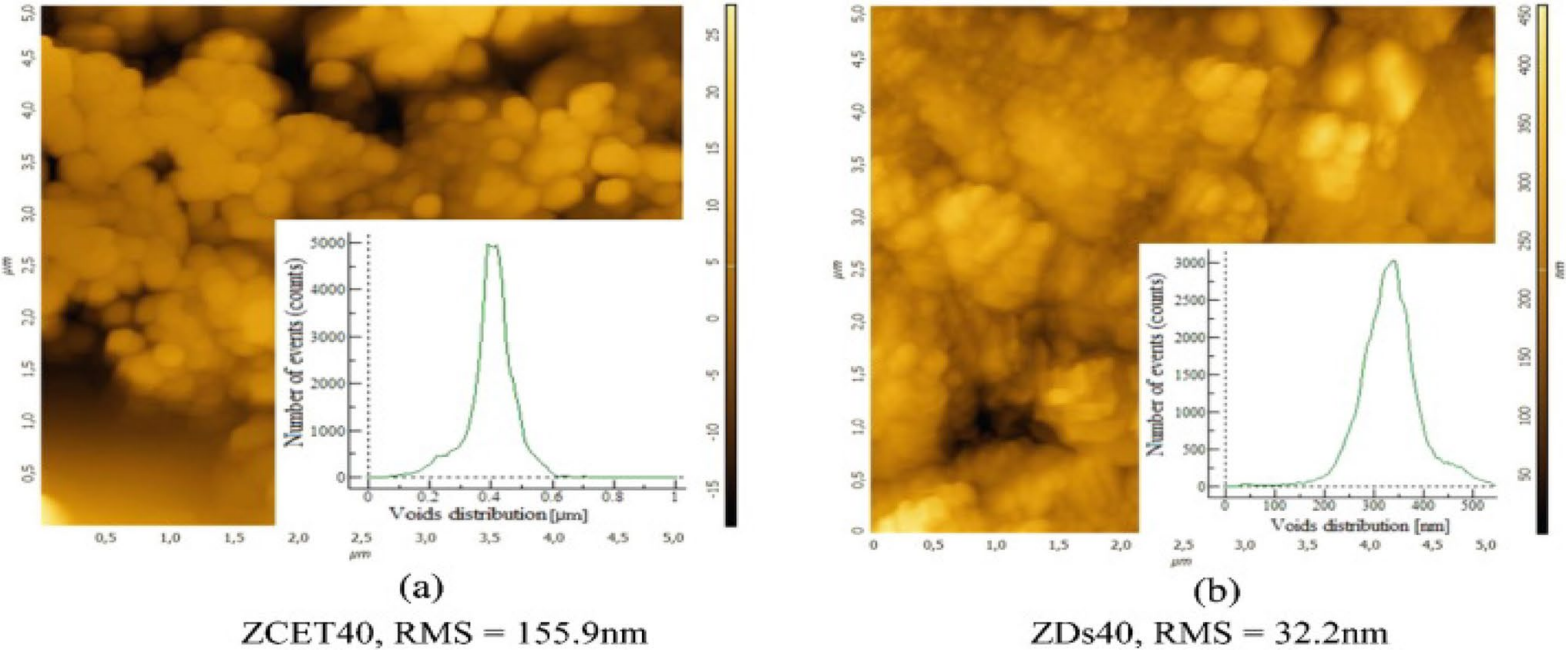
AFM topography, average roughness, and pore distribution: **a** ZCET40; **b** ZDs40 (Visa 2016)

The surface topography, average roughness value, and pore distribution of the synthesized zeolites are measured by atomic force microscopy (AFM) (Visa 2016). It reveals the details of the crystal growth mechanism of one-dimensional nanoporous aluminosilicate structures (Fig. 15).

### XRF analysis

Elements in a solid sample can be detected by X-ray fluorescence (XRF) spectrometry, a multielement detection method. There are two types of approaches to the analytical use of XRF spectrometry: wavelength-dispersive and energy-dispersive XRF spectrometry. Although wavelength spectrometry is generally more sensitive, it has more expensive equipment requirements (Suárez 2001).

Elemental analysis of raw materials before treatment can be performed with wavelength-dispersive X-ray fluorescence (XRF) spectrometry (Tan et al. 2011). It is an effective technique for analyzing co-destruction precipitates by XRF spectrometry for the quantitative determination of SiO_2_ and Al_2_O_3_ and determining their suitability for use as raw materials for hydrothermal zeolite synthesis (Somerset et al. 2004).

This technique is also applied to characterize synthesized zeolites. The ionic exchange capacity, that is, the CEC value, of synthetic zeolites can be determined using XRF and previously described EPMA techniques. The main difference between the two spectroscopic techniques is the sample excitation mode and size. Photons are excited in the XRF technique and electrons are excited in the EPMA technique (Galván et al. 2009). XRF and EPMA techniques can be considered innovations as they allow to analyze the sample directly. The use of these methods is the preferred technique as they are not destructive to the structure, provide the opportunity to analyze with small sample amounts, and have other advantageous features such as short total analysis time (Galván et al. 2009).

### ICP-MS

ICP-MS technique can be used to more clearly understand the composition of zeolite filtrates after synthesis and the types of trace and heavy metals in the structure. This technique determines which elements remain in the structure of synthesized zeolites. A study is being carried out to determine the heavy metals that may be encapsulated in zeolitic materials during synthesis and to determine the heavy metal content of solid zeolite materials synthesized by the acid leaching method (Somerset et al. 2004).

### BET

Surface area is one of the most important properties for the characterization of microporous materials, especially zeolites. In general, experimental surface areas are characterized by the Langmuir surface area or the BET surface area technique obtained using nitrogen adsorption isotherms at 77 K. The choice between the two surface areas depends on whether the pores in the structure support multilayer (BET) or single-layer only (Langmuir) adsorption (Bae et al. 2010).

The BET technique is used to measure the external surface areas of zeolites. Surface areas are calculated from nitrogen isotherms using the BET equation. The shape of adsorption isotherms provides qualitative information about adsorption processes and the surface area available for adsorbate property.

### Sorption tests

In adsorption studies of synthesized zeolites, studies are carried out under different experimental conditions in order to ensure the highest amount of removal. Experimental parameters are generally shaking speed, pH, contact time, adsorbent dosage, and initial concentration of the solution for removal study. Interpretations are made by evaluating the isotherm kinetics obtained as a result of the study. All experiments are repeated several times under different conditions and average data are reported. “% Removal” and efficiency for the substance removed by adsorption study are calculated with the equations given below (Javadian et al. 2015).

1 $$\text{Removal efficiency} \left(\%\right)=({C}_{\text{i}}-{C}_{t})/{C}_{\text{i}}\times 100$$

2 $${q}_{t}=({C}_{\text{i}}-{C}_{t})\times V/M$$

In the equation, “*C*_i_” is the initial concentration (mg∙g^−1^), “*C*_*t*_” is the concentration value at any time “*t*,” “*q*_*t*_” is the amount of adsorbent per amount of adsorbent (mg g^−1^), *V* is the solution volume, and *M* is the adsorbate mass. expresses (Fukuda et al. 2023).

### Adsorption kinetics and isotherms

For zeolite samples, graphs of change in adsorbed amount *q*_*t*_ with contact time *t* are created. In analyzing the obtained adsorption kinetics, several commonly used kinetic models (pseudo-first-order kinetic model, pseudo-second-order kinetic model, Elovich model, and particle model) were used, as expressed in Eqs. (2), (3), (4), and (5), respectively. Intra-diffusion model is adopted.

3 $${q}_{t}={q}_{e}(1-{e}^{-{k}_{1}t})$$

4 $${q}_{t}=\frac{{k}_{2}{{q}_{e}}^{2}t}{1+{k}_{2}{q}_{e}t}$$

5 $${q}_{t}=\frac{1}{\beta }\text{ln}\left(\alpha \beta t\right)$$

6 $${q}_{t}={k}_{i}{t}^{0.5}+c$$

Here, *k* (h^–1^) and *k* (g/(mg·h)) are the rate constants of adsorption. *α* (mg/(g·h)) and *β* (g/mg) are the initial adsorption rate and the contrast related to the size of the surface coverage and activation energy for chemisorption, respectively. *k* (mg/(g·h^−0.5^)) is the rate constant and *c* (mg/g) is the constant. Equations (2), (3), and (4) are transformed into linear forms:

7 $$\text{ln}\left({q}_{e}-{q}_{t}\right)=\text{ln}{q}_{e}-{k}_{1}t$$

8 $$\frac{t}{{q}_{t}}=\frac{1}{{k}_{2}{{q}_{e}}^{2}}+\frac{t}{{q}_{e}}$$

9 $${q}_{t}=\frac{1}{\beta }\text{ln}\left(\alpha \beta \right)+\frac{1}{\beta }\text{ln}t$$

The kinetic model that fits the adsorption mechanism is determined with the linear graphs obtained as a result of the equations (Benjelloun et al. 2021).

Fukuda et al. (2023) synthesized Na-P1 zeolite by utilizing silica waste obtained from the quartz glass production process as an absorbent for the removal of Cs^+^ and Sr^2+^ from aqueous solutions. They investigated the adsorption kinetics and isotherms of GIS-NaP1 zeolite, which they developed by hydrothermal synthesis method for the removal of radioactive Cs^+^ and Sr^2+^ from environments, and evaluated the adsorption performance (Fukuda et al. 2023). In this study, they analyzed the adsorption isotherms using the Langmuir, Freundlich, Temkin, and Dubinin-Radushkevich models defined by Eqs. (10), (11), (12), and (13), respectively.

10 $${q}_{e}=\frac{{q}_{m}{K}_{L}{C}_{e}}{1+{K}_{L}{C}_{e}}$$

11 $${q}_{e}={K}_{F}{{C}_{e}}^{1/n}$$

12 $${q}_{e}={q}_{T}(\text{ln}({A}_{T}{C}_{e})$$

13 $${q}_{e}={q}_{m}\text{exp}[-\frac{1}{2{E}^{2}}{\{RT\text{ln}(1+\frac{1}{{C}_{e}})\}}^{2}]$$

*q* (mg/g) refers to the adsorption capacity of the adsorbent, *K* (L/mg) refers to the Langmuir constant, and *K* (L/mg) and *n* [ −] refers to the Freundlich constants. *A* (L/mg) and *q* (mg/g) express the adsorption equilibrium constant of the substance dissolved on the solid surface and the surface adsorption capacity per unit binding energy, respectively. *R* (J/(mol·K)) is the gas constant, *T* (K) is the absolute temperature, and *E* (J/mol) is the adsorption energy (X. Chen et al. 2022a, b; Javadian et al. 2015). After the operations, Eqs. (9)–(12) are converted into linear forms (Table 8).

14 $$\frac{{C}_{e}}{{q}_{e}}=\frac{1}{{q}_{m}{K}_{L}}+\frac{{C}_{e}}{{q}_{m}}$$

15 $$\text{ln }{q}_{e} =\text{ln}{K}_{F}+\frac{1}{n}\text{ln}{C}_{e}$$

16 $${q}_{e}= {q}_{T}\text{ln}{A}_{T}+{q}_{T}\text{ln}{C}_{e}$$

17 $$\text{ln }{q}_{e}=\text{ln}{q}_{m}-{\left(\frac{RT}{\sqrt{2E}}\right)}^{2}{\{\text{ln}\left(1+\frac{1}{{C}_{e}}\right)\}}^{2}$$

**Fig. 16:**
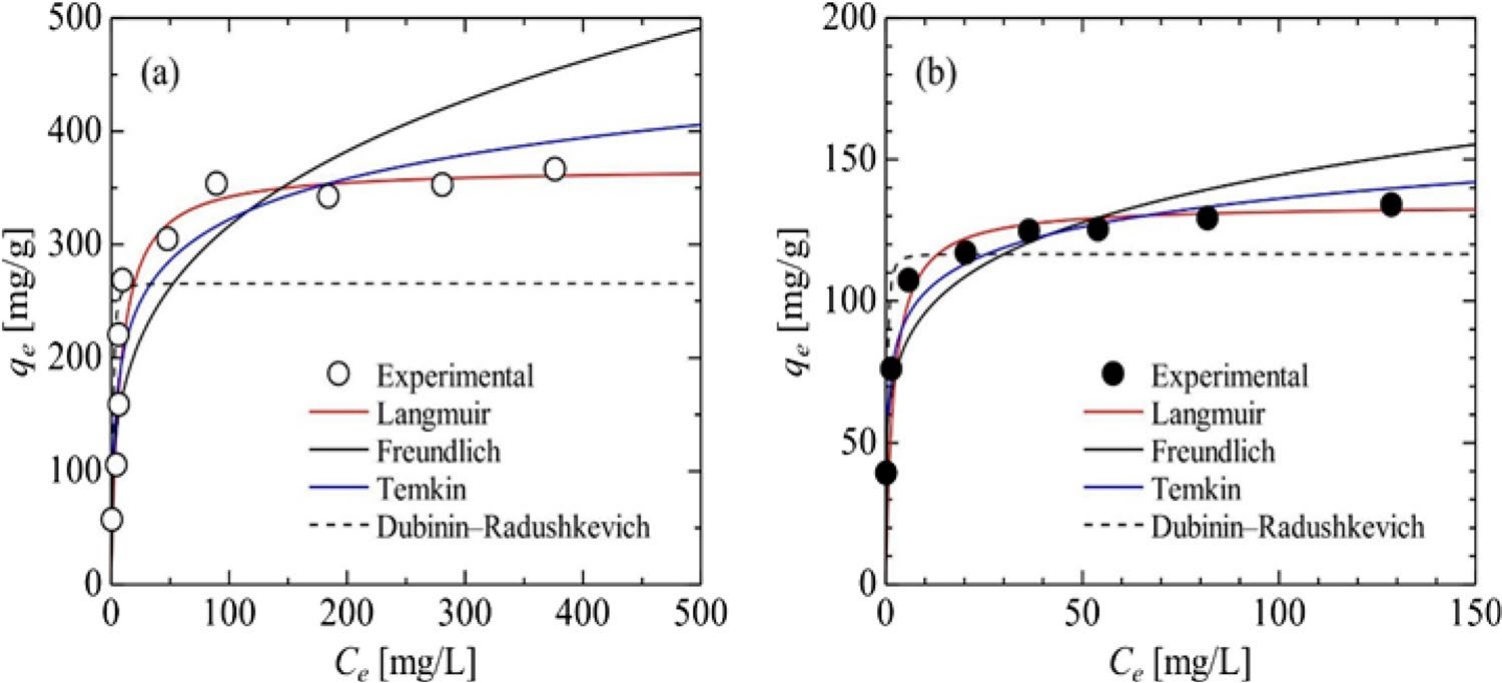
Adsorption isotherm of zeolite sample for **a** Cs^+^ and **b** Sr.^2+^ (Fukuda et al. 2023)

In their study, Fukuda et al. (2023) plotted the experimental data for Cs^+^ and Sr^2+^ linearly, according to Eqs. (14)–(17). As shown in Fig. 16, the graphs fit the Langmuir equation adsorption isotherm data well, indicating that the adsorption was homogeneous monolayer adsorption. When the adsorption behavior of GIS-NaP1 zeolite was examined, it was proven that it had relatively small rate constants but high adsorption capacity and was suitable for Cs^+^ and Sr^2+^ adsorption (Fukuda et al. 2023).

**Table 8:** Kinetics of sorption

Kinetic model		Ref
Morris–Weber	${q}_{t}={K}_{id}{(t)}^{0.5}+C$	(Chen et al. 2022a, b)
Lagergren	$\text{ln}\left({q}_{e}-{q}_{t}\right)=\text{ln}{q}_{e}-Kt$	(Kwon et al. 2020)
Pseudo-second order	${q}_{t}={k}_{2}{{q}_{e}}^{2}t/1+{k}_{2}{q}_{e}t$	(Al-Jubouri et al. 2023; Cheng et al. 2021a, b; Huang et al. 2023)
Elovich	${q}_{t}=1/\beta \text{ ln}(\alpha \beta )+1/\beta ln t$	(Al-Jubouri et al. 2023)
Isotherm model		
Langmuir	${q}_{e}={q}_{m}{K}_{L}{C}_{e}/(1+{K}_{L}{C}_{e})$ ${R}_{L}=1/1+{K}_{L}{C}_{i}$	(Fukuda et al. 2023; Xiang et al. 2023)
Freundlich	${q}_{e}={K}_{F}{({C}_{e})}^{1/n}$ $\text{log}\left({q}_{e}\right)=\text{log}\left({K}_{F}\right)+(1/n)\text{log }({C}_{e})$	(Cheng et al. 2021a, b)
Tempkin	${q}_{e}=B\text{ ln }({K}_{T}{C}_{e})$ ${q}_{e}=B\text{ ln }{K}_{T}+B\text{ ln}{ C}_{e}$	(Zhang et al. 2011)
Dubinin-Radushkevick	$\text{ln}{q}_{e}=\text{ln}{q}_{m}-\beta {\varepsilon }^{2}$ $\varepsilon =RT\text{ln}[(1+1/{C}_{e})]$ $E=1/\surd 2\beta$	(Javadian et al. 2015)

### Cation exchange capacity

The cation exchange capacity (CEC) of a zeolite depends on the chemical composition, structure, and cation nature of the zeolite. CEC is determined depending on the number of interchangeable positions, and these values vary depending on the SiO_2_/Al_2_O_3_ ratio (Galván et al. 2009).

The process of determining cation exchange capacity (CEC) involves the balance change of ammonium (NH_4_^+^) ions and their subsequent exchange with (K^+^) ions. The assumption in this process is that NH_4_^+^ ions charge all active acid sites and are then replaced by K^+^ ions through the ion exchange process (Eq. (18).

18 $$\text{CEC}\left(\frac{\text{meq}}{100\text{g}}\right)=\frac{\left(\frac{{C}_{\text{N}-{\text{NH}}_{3}}\times {V}_{\text{sample}}}{{M}_{\text{sample}}}\right)}{{M}_{\text{r}}}\div 10$$

In the equation, “*C*” refers to the concentration of ammonium ions in the extract solution (mg/L), “*V*_sample_” refers to the solution volume (mL), “*M*_sample_” refers to the zeolite adsorbent mass (g), and “*M*_r_” refers to the molecular mass of NH_4_^+^. CEC is determined following ASTM Standard Test Method D7503-18 (Zijun et al. 2021) (Table 9).

**Table 9:** Cation exchange capacities of zeolite types synthesized from different types of raw materials

Si/Al source	Synthesis method	Zeolite type	Cation exchange capacity	Ref
Fly ash	Conventional hydrothermal method	Zeolite A	237.3 mmol/100 g	(Fan et al. 2023)
Fly ash	Alkali fusion hydrothermal method	Zeolite P	497.01 mg/g	(Yang et al. 2023)
Ca-Bentonite	Alkali fusion hydrothermal method	Zeolite HS	282 mg/g	(Mamaghani et al. 2022)
Foundry dust	Alkali fusion hydrothermal method	Zeolite NaA	37.81 mg/g	(Wang et al. 2023)
Low-grade kaolin	Conventional hydrothermal method	Zeolite A	250 mg/g	(Ayele et al. 2016)
Low-grade kaolin	Alkali fusion hydrothermal method	Zeolite A	300 mg/g	(Ayele et al. 2016)

### Factors affecting adsorption capacity of zeolites

#### Time contact effect

One of the variables affecting adsorption processes is adsorption time. It can be described through so-called first- and second-order kinetic models (Alabbad 2021).

19 $$\text{ln}\left({Q}_{e}-{Q}_{t}\right)=\text{ln}{Q}_{e}-{k}_{1}t$$

20 $$\frac{t}{{Q}_{t}}=\frac{1}{{k}_{2}{{Q}_{e}}^{2}}+\frac{t}{{Q}_{e}}$$

#### Effect of pH

In adsorption processes, solution pH is extremely important to evaluate the behavior of adsorbent materials in solution. By analyzing the pH value of the material at the zero load point (pH_pzc_), the pH value at which the adsorbent surface remains neutral is determined. This expression enables the evaluation of the interactions between the adsorbent and the adsorbate by estimating the surface functional group ionization. When the pH of the solution is above the pHpzc value (zero charge point), the adsorbent surface carries a negative charge, in which case it shows a tendency to affinity towards cations (positively charged species). Otherwise, when the pH of the solution is lower than pHpzc, the surface becomes positively charged and shows affinity for negative charges (Dias et al. 2023).

In a study, it was observed that due to the nature of the cationic dye (RhB), the removal of dye molecules becomes easier around pH > 8 due to the increase in electrostatic interactions between the zeolite surface and RhB. A decrease in the pHZCP of the cobalt-doped zeolite was observed, and this was interpreted as resulting from the entry of cobalt oxide into the crystal structure. This situation leads to an increase in electrostatic interactions such as protonation of Co^2+^ ions in the alkaline pH state (Pedebos et al. 2024).

#### Effect of adsorbent dosage

In adsorption studies, the effect of adsorption dosage on adsorption capacity is examined. Studies show that the adsorbate level increases with the increase in adsorbent dosage. It is observed that as the adsorbent substance is added to a certain level, the increase in the removal amount is rapid up to a certain level, then slows down and is completely adsorbed (Javadian et al. 2015).

### The thermodynamics of adsorption on zeolites

The adsorption process is analyzed by using the Van’t Hoff equation, changes in Gibb’s free energy (Δ*G*), changes in enthalpy (Δ*H*°), and entropy (Δ*S*°) with Eqs. (18)–(20). The Van’t Hoof equation is used to express the changes in the equilibrium constant with temperature change (Lima et al. 2019).

21 $$\Delta {G}^{0}=-RT\text{ln} ({{K}_{e}}^{0})$$

22 $$\Delta {G}^{0}=\Delta {H}^{0}-T.\Delta {S}^{0}$$

23 $$\text{ln}\left({{K}_{e}}^{0}\right)=\frac{-\Delta {H}^{0}}{R}.\frac{1}{T}+\frac{\Delta {S}^{0}}{R}$$

Here, $\Delta {G}^{0}$ (J/mol), $\Delta {S}^{0}$ (J/(mol·K)), $\Delta {H}^{0}$ [J/mol] and ${{K}_{e}}^{0}$ [ −] are Gibbs free energy, standard entropy, standard enthalpy and thermodynamic equilibrium constant, respectively.

$\text{ln}\left({{K}_{e}}^{0}\right)$ *to* $\frac{1}{T}$ The graph is created and while the intersection point expresses the change in entropy (∆*S*^0^), the slope expresses the change in enthalpy ∆*H*^0^. By expressing adsorption isotherms at different temperatures, the equilibrium constant of adsorption can be calculated accurately. The most compatible model expressed at different temperatures is selected and made dimensionless so that it can be applied in the Van’t Hoff equation, and *K*_*L*_ is determined (Lima et al. 2019) (Table 10). If ∆*H*^0^ is positive, the zeolite is endothermic; otherwise, the zeolite is exothermic. The positive value of ∆*S*^0^ indicates that the affinity of cations towards zeolite is high and the randomness at the solid-solution interface increases.

**Table 10:** Thermodynamics of zeolites

Zeolite	Adsorbates	$\Delta {H}^{0}$ $[\text{kJ}/\text{mol}]$	$\Delta {S}^{0}$ $[\text{J}/(\text{mol}\cdot \text{K})]$	$\Delta {G}^{0}$ $[\text{kJ}/\text{mol}]$	$Qe$(mg/g)	Ref
Zeolite (NZ)	Direct Yellow 50	− 11.814	− 26.5123	− 4.01317	83.84	(Alabbad 2021)
ZO-Co	Rodamin B	55.65	20.39	− 5.47	132.56	(Pedebos et al. 2024)
ZNSC	Cd(II) ions	7.80	54.46	− 8.43	324.3	(Zhang et al. 2023)
Zeolite-Ag_2_S	H_g_Cl_2_	− 10.33	0.027	− 19.13	268	(Jena et al. 2023)

## Conclusion

In this review, recent developments in the conversion of industrial solid wastes into zeolites, a value-added product, have been evaluated. Crystallization methods of zeolites are among the current research topics. Compared to natural zeolites, synthetic zeolites exhibit highly efficient adsorption–desorption and ion exchange capability with uniform pore sizes, molecular sieve effect, high cation exchange capacity, and hydrothermal stability. Due to these properties, interest in artificial zeolites has increased today.

In recent years, the elimination of environmental problems has become the center of attention of scientists. Zeolites are functional materials synthesized for this purpose.

Zeolites, which are called molecular sieves, have applications in many areas such as fuel cells; thermal energy storage, carbon capture, CO_2_ storage, air pollution remediation, removal of pollutants such as oil, heavy metals, radioactive wastes, dyes, oils and salts from water, biomedical applications thanks to their porous structure, acting as catalysts thanks to their Brønsted acid sites, Lewis acid sites and multifunctional active sites.

The adsorption isotherms, kinetics and thermodynamics of the removal of heavy metals and dyes from wastewater by zeolites were investigated and the characterization techniques of synthetic zeolites were evaluated in detail. As a result, it was determined that adsorption time, pH and initial concentration change the adsorption capacity of zeolites due to the change in their surface properties.

Hydrothermal synthesis method, which is the traditional zeolite synthesis method, has disadvantages such as long reaction time and inability to reach the desired purity in the synthesized zeolites. The hydrothermal synthesis method can be improved by supporting techniques such as alkaline fusion, ultrasonic and microwave support. Alkaline fusion support provides higher conversion and purity, the microwave method significantly accelerates the synthesis time, and ultrasonic support shortens the zeolite synthesis time and reduces energy consumption. However, large-scale applications of these techniques are lacking. It is expected that making such methods available for wider industrial use would be highly beneficial from an environmental perspective. It will be an effective step towards achieving a green technology.

## Data Availability

The data that support the findings of this study are available from the corresponding author, upon reasonable request.

## References

[CR1] Abbou B, Lebkiri I, Ouaddari H, El Amri A, Achibat FE, Kadiri L, Ouass A, Lebkiri A, Rifi EH (2023). Improved removal of methyl orange dye by adsorption using modified clay: combined experimental study using surface response methodology. Inorg Chem Commun.

[CR2] Abdelrahman EA (2018). Synthesis of zeolite nanostructures from waste aluminum cans for efficient removal of malachite green dye from aqueous media. J Mol Liq.

[CR3] Abdullahi M, Ojelade GO, Auta SM (2017). Modified water-cement ratio law for compressive strength of rice husk ash concrete. Niger J Technol.

[CR4] Adel M, Ahmed MA, Elabiad MA, Mohamed AA (2022). Removal of heavy metals and dyes from wastewater using graphene oxide-based nanomaterials: a critical review. Environ Nanotechnol Monit Manag.

[CR5] Ahmaruzzaman M (2010). A review on the utilization of fly ash. Prog Energy Combust Sci.

[CR6] Alabbad EA (2021). Efficacy assessment of natural zeolite containing wastewater on the adsorption behaviour of Direct Yellow 50 from; equilibrium, kinetics and thermodynamic studies. Arab J Chem.

[CR7] Aldahri T, Behin J, Kazemian H, Rohani S (2016). Synthesis of zeolite Na-P from coal fly ash by thermo-sonochemical treatment. Fuel.

[CR8] Al-Jubouri SM, Al-Jendeel HA, Rashid SA, Al-Batty S (2023). Green synthesis of porous carbon cross-linked Y zeolite nanocrystals material and its performance for adsorptive removal of a methyl violet dye from water. Microporous Mesoporous Mater.

[CR9] Amin KF, Gulshan F, Asrafuzzaman FNU, Das H, Rashid R, Manjura Hoque S (2023). Synthesis of mesoporous silica and chitosan-coated magnetite nanoparticles for heavy metal adsorption from wastewater. Environ Nanotechnol Monit Manag.

[CR10] Anderson A, Anbarasu A, Pasupuleti RR, Manigandan S, Praveenkumar TR, Aravind Kumar J (2022). Treatment of heavy metals containing wastewater using biodegradable adsorbents: a review of mechanism and future trends. Chemosphere.

[CR11] Asaithambi P, Govindarajan R, Busier Yesuf M, Selvakumar P, Alemayehu E (2020). Enhanced treatment of landfill leachate wastewater using sono(US)-ozone(O3)–electrocoagulation(EC) process: role of process parameters on color, COD and electrical energy consumption. Process Saf Environ Prot.

[CR12] Ayele L, Pérez-Pariente J, Chebude Y, Díaz I (2016). Conventional versus alkali fusion synthesis of zeolite A from low grade kaolin. Appl Clay Sci.

[CR13] Bae YS, Yazaydın AO, Snurr RQ (2010). Evaluation of the BET method for determining surface areas of MOFs and zeolites that contain ultra-micropores. Langmuir.

[CR14] Barakat MA (2011). New trends in removing heavy metals from industrial wastewater. Arab J Chem.

[CR15] Barrer RM, Marcilly C (1970) Hydrothermal chemistry of silicates. Part XV. Synthesis and nature of some salt-bearing aluminosilicates. J Chem Soc: Inorg Phys Theor Chem 2735–2745. 10.1039/J19700002735

[CR16] Batubara AS, Adress Hasan HM, Abel Moniem MA, Masoud MS, Mostafa AER, Gamal M, Elsayed MA (2023). Usage of natural wastes from animal and plant origins as adsorbents for the removal of some toxic industrial dyes and heavy metals in aqueous media. Journal of Water Process Engineering.

[CR17] Bechtold T, Mussak R, Mahmud-Ali A, Ganglberger E, Geissler S (2006). Extraction of natural dyes for textile dyeing from coloured plant wastes released from the food and beverage industry. J Sci Food Agric.

[CR18] Behin J, Bukhari SS, Dehnavi V, Kazemian H, Rohani S (2014). Using coal fly ash and wastewater for microwave synthesis of LTA zeolite. Chem Eng Technol.

[CR19] Belviso C, Cavalcante F, Lettino A, Fiore S (2009). Zeolite synthesised from fused coal fly ash at low temperature using seawater for crystallization. Coal Combustion and Gasification Products.

[CR20] Benjelloun M, Miyah Y, Akdemir Evrendilek G, Zerrouq F, Lairini S (2021). Recent advances in adsorption kinetic models: their application to dye types. Arab J Chem.

[CR21] Bensafi B, Chouat N, Djafri F (2023). The universal zeolite ZSM-5: structure and synthesis strategies. Rev Coord Chem Rev.

[CR22] Bonakdarpour B, Vyrides I, Stuckey DC (2011). Comparison of the performance of one stage and two stage sequential anaerobic–aerobic biological processes for the treatment of reactive-azo-dye-containing synthetic wastewaters. Int Biodeterior Biodegradation.

[CR23] Bradley SA, Broach RW, Mezza TM, Prabhakar S, Sinkler W (2010) Zeolite characterization. Zeolites Ind Sep Catal 85–171. 10.1002/9783527629565.CH4

[CR24] Bunmai K, Osakoo N, Deekamwong K, Rongchapo W, Keawkumay C, Chanlek N, Prayoonpokarach S, Wittayakun J (2018). Extraction of silica from cogon grass and utilization for synthesis of zeolite NaY by conventional and microwave-assisted hydrothermal methods. J Taiwan Inst Chem Eng.

[CR25] Campbell LS, Charnock J, Dyer A, Hillier S, Chenery S, Stoppa F, Henderson CMB, Walcott R, Rumsey M (2016). Determination of Zeolite-Group Mineral Compositions by Electron Probe Microanalysis.

[CR26] Cao C, Xuan W, Yan S, Wang Q (2023). Zeolites synthesized from industrial and agricultural solid waste and their applications: a review. J Environ Chem Eng.

[CR27] Chanda R, Islam MS, Biswas BK (2023). N and P removal from wastewater using rice husk ash-derived silica-based Fe-ZSM-5 zeolite. Cleaner Engineering and Technology.

[CR28] Chang JI, Tsai JJ, Wu KH (2006). Composting of vegetable waste.

[CR29] Chen Y, Yang X (2022). Molecular simulation of layered GO membranes with amorphous structure for heavy metal ions separation. J Membr Sci.

[CR30] Chen Q, Yao Y, Li X, Lu J, Zhou J, Huang Z (2018). Comparison of heavy metal removals from aqueous solutions by chemical precipitation and characteristics of precipitates. Journal of Water Process Engineering.

[CR31] Chen D, Tang Q, Deng W, Chaianansutcharit S, Guo L (2022). Comparative studies on the toluene sorption performance over silicalite-1 zeolites with different morphologies. Microporous Mesoporous Mater.

[CR32] Chen X, Hossain MF, Duan C, Lu J, Tsang YF, Islam MS, Zhou Y (2022). Isotherm models for adsorption of heavy metals from water - a review. Chemosphere.

[CR33] Chen Z, Song G, Li C, Chen W, Li Z, Kawi S (2023). Coal fly ash to Y zeolite of great purity and crystallinity: a new and green activation method of combined in situ microwave and ultrasound. Solid State Sci.

[CR34] Cheng TH, Sankaran R, Show PL, Ooi CW, Liu BL, Chai WS, Chang YK (2021). Removal of protein wastes by cylinder-shaped NaY zeolite adsorbents decorated with heavy metal wastes. Int J Biol Macromol.

[CR35] Cheng Y, Xu L, Liu C (2021). NaP1 zeolite synthesized via effective extraction of Si and Al from red mud for methylene blue adsorption. Adv Powder Technol.

[CR36] Chipasa KB (2003). Accumulation and fate of selected heavy metals in a biological wastewater treatment system. Waste Manage.

[CR37] Chu S, Liu C, Feng X, Wu H, Liu X (2023). Aromatic polymer dual-confined magnetic metal-organic framework microspheres enable highly efficient removal of dyes, heavy metals, and antibiotics. Chem Eng J.

[CR38] Conte N, Gómez JM (2024). Improving the sorption properties of mesoporous carbons for the removal of cobalt, nickel and manganese from spent lithium-ion batteries effluent. Sep Purif Technol.

[CR39] Cundy CS, Cox PA (2005). The hydrothermal synthesis of zeolites: precursors, intermediates and reaction mechanism. Microporous Mesoporous Mater.

[CR40] Daer S, Kharraz J, Giwa A, Hasan SW (2015). Recent applications of nanomaterials in water desalination: a critical review and future opportunities. Desalination.

[CR41] Dapsens PY, Mondelli C, Pérez-Ramírez J (2015). Design of Lewis-acid centres in zeolitic matrices for the conversion of renewables. Chem Soc Rev.

[CR42] Darmansyah D, You SJ, Wang YF (2023). Advancements of coal fly ash and its prospective implications for sustainable materials in Southeast Asian countries: a review. Renew Sustain Energy Rev.

[CR43] Dewajani H, Zamrudy W, Irfin Z, Ningtyas D, Mujibur Ridlo N (2023). Utilization of Indonesian sugarcane bagasse into bio asphalt through pyrolysis process using zeolite-based catalyst. Materials Today: Proceedings.

[CR44] Dias R, Daam MA, Diniz M, Maurício R (2023). Drinking water treatment residuals, a low-cost and environmentally friendly adsorbent for the removal of hormones - a review. Journal of Water Process Engineering.

[CR45] Dong L, Hou L, Wang Z, Gu P, Chen G, Jiang R (2018). A new function of spent activated carbon in BAC process: removing heavy metals by ion exchange mechanism. J Hazard Mater.

[CR46] El-habacha M, Miyah Y, Lagdali S, Mahmoudy G, Dabagh A, Chiban M, Sinan F, Iaich S, Zerbet M (2023). General overview to understand the adsorption mechanism of textile dyes and heavy metals on the surface of different clay materials. Arab J Chem.

[CR47] ElMekawy A, Srikanth S, Bajracharya S, Hegab HM, Nigam PS, Singh A, Mohan SV, Pant D (2015). Food and agricultural wastes as substrates for bioelectrochemical system (BES): the synchronized recovery of sustainable energy and waste treatment. Food Res Int.

[CR48] Fan M, Dai D, Huang B (2012). Fourier transform infrared spectroscopy for natural fibres. Fourier Transform-Mater Anal.

[CR49] Fan Y, Huang R, Liu Q, Cao Q, Guo R (2023). Synthesis of zeolite A from fly ash and its application in the slow release of urea. Waste Manage.

[CR50] Finish N, Ramos P, Borojovich EJC, Zeiri O, Amar Y, Gottlieb M (2023). Zeolite performance in removal of multicomponent heavy metal contamination from wastewater. J Hazard Mater.

[CR51] Flores CG, Schneider H, Dornelles JS, Gomes LB, Marcilio NR, Melo PJ (2021). Synthesis of potassium zeolite from rice husk ash as a silicon source. Clean Eng Technol.

[CR52] Forgacs E, Cserháti T, Oros G (2004). Removal of synthetic dyes from wastewaters: a review. Environ Int.

[CR53] Fu F, Wang Q (2011). Removal of heavy metal ions from wastewaters: a review. J Environ Manage.

[CR54] Fujii S, Nakagaki T, Kanematsu Y, Kikuchi Y (2022). Prospective life cycle assessment for designing mobile thermal energy storage system utilizing zeolite. J Clean Prod.

[CR55] Fukuda M, Onizuka T, Tokumaru H, Horikoshi H, Iwasaki T (2023). Synthesis of NaP1 zeolite from silica waste as an absorbent for the removal of Cs+ and Sr2+ from aqueous solution. Chem Eng Res Des.

[CR56] Fyfe CA, Grondey H, Feng Y, Kokotailo GT (1990) Natural-abundance two-dimensional 29Si MAS NMR investigation of the three-dimensional bonding connectivities in the zeolite catalyst ZSM-5. J Am Chem Soc 112:8812–8820. https://pubs.acs.org/sharingguidelines

[CR57] Galván V, Galván G, Torres Deluigi M, Mentasty L, De Vito I, Riveros JA (2009). Comparison between XRF and EPMA applied to study the ionic exchange in zeolites. X-Ray Spectrom.

[CR58] Ghasemi H, Afshang M, Gilvari T, Aghabarari B, Mozaffari S (2023). Rapid and effective removal of heavy metal ions from aqueous solution using nanostructured clay particles. Results Surf Interfaces.

[CR59] González-Muñoz MJ, Rodríguez MA, Luque S, Álvarez JR (2006). Recovery of heavy metals from metal industry waste waters by chemical precipitation and nanofiltration. Desalination.

[CR60] Gul A, Ma’amor A, Khaligh NG, Muhd Julkapli N (2022). Recent advancements in the applications of activated carbon for the heavy metals and dyes removal. Chem Eng Res Des.

[CR61] Hassan H, Hameed BH (2023). Green hydroxyapatite-zeolite catalyst derived from steel waste as an effective catalyst for the hydrocarbon production via co-catalytic pyrolysis of sugarcane bagasse and high-density polyethylene. Catal Commun.

[CR62] He X, Yao B, Xia Y, Huang H, Gan Y, Zhang W (2020). Coal fly ash derived zeolite for highly efficient removal of Ni2+ inwaste water. Powder Technol.

[CR63] Hosseinkhani O, Hamzehlouy A, Dan S, Sanchouli N, Tavakkoli M, Hashemipour H (2023). Graphene oxide/ZnO nanocomposites for efficient removal of heavy metal and organic contaminants from water. Arab J Chem.

[CR64] Huang X, LangLi LJS, Poon CS (2023). Synthesis of Na-A zeolite loaded bentonite and its application for removal of Cu(II) from aqueous solutions. J Water Process Eng.

[CR65] Hussar K, Teekasap S, Somsuk N (2011). Synthesis of zeolite A from by-product of aluminum etching process: effects of reaction temperature and reaction time on pore volume. Am J Environ Sci.

[CR66] Hussein TK, Jasim NA (2021). A comparison study between chemical coagulation and electro-coagulation processes for the treatment of wastewater containing reactive blue dye. Mater Today Proc.

[CR67] Iqbal A, Sattar H, Haider R, Munir S (2019). Synthesis and characterization of pure phase zeolite 4A from coal fly ash. J Clean Prod.

[CR68] Iwuozor KO, Ighalo JO, Emenike EC, Ogunfowora LA, Igwegbe CA (2021). Adsorption of methyl orange: a review on adsorbent performance. Curr Res Green Sustain Chem.

[CR69] Jacobs PA, Santen RA. van (Rutger A) (1989) Zeolites : facts, figures, future : proceedings of the 8th international zeolite conference, Amsterdam, the Netherlands, July 10–14, 1989. Part A 49:1466

[CR70] Javadian H, Ghorbani F, TayebiAsl HASH (2015). Study of the adsorption of Cd (II) from aqueous solution using zeolite-based geopolymer, synthesized from coal fly ash; kinetic, isotherm and thermodynamic studies. Arab J Chem.

[CR71] Jena KK, Reddy KS, Karanikolos GN, Choi DS (2023). l-Cysteine and silver nitrate based metal sulfide and zeolite-Y nano adsorbent for efficient removal of mercury (II) ion from wastewater. Appl Surf Sci.

[CR72] Ji W, Zhang S, Zhao P, Zhang S, Feng N, Lan L, Zhang X, Sun Y, Li Y, Ma Y (2020) Green synthesis method and application of NaP zeolite prepared by coal gasification coarse slag from Ningdong, China. Appl Sci (Switzerland) 10(8). 10.3390/APP10082694

[CR73] Jin Y, Li L, Liu Z, Zhu S, Wang D (2021). Synthesis and characterization of low-cost zeolite NaA from coal gangue by hydrothermal method. Adv Powder Technol.

[CR74] Jin Y, Xu Q, Zheng F, Lu J (2023). Enhancement in CO2 adsorption by zeolite synthesized from co-combustion ash of coal and rice husk modified with lithium ion. J Energy Inst.

[CR75] Kafle BP (2020) Introduction to nanomaterials and application of UV–visible spectroscopy for their characterization. Chem Anal Mater Charact Spectrophotometry 147–198. 10.1016/B978-0-12-814866-2.00006-3

[CR76] Katheresan V, Kansedo J, Lau SY (2018). Efficiency of various recent wastewater dye removal methods: a review. J Environ Chem Eng.

[CR77] Khaleque A, Alam MM, Hoque M, Mondal S, Haider JB, Xu B, Johir MAH, Karmakar AK, Zhou JL, Ahmed MB, Moni MA (2020). Zeolite synthesis from low-cost materials and environmental applications: a review. Environ Adv.

[CR78] Khan FSA, Mubarak NM, Tan YH, Khalid M, Karri RR, Walvekar R, Abdullah EC, Nizamuddin S, Mazari SA (2021). A comprehensive review on magnetic carbon nanotubes and carbon nanotube-based buckypaper for removal of heavy metals and dyes. J Hazard Mater.

[CR79] Khan MD, Singh A, Khan MZ, Tabraiz S, Sheikh J (2023). Current perspectives, recent advancements, and efficiencies of various dye-containing wastewater treatment technologies. J Water Proc Eng.

[CR80] Khan NA, Yoo DK, Lee S, Kim TW, Kim CU, Jhung SH (2023). Microwave-assisted rapid synthesis of nanosized SSZ-13 zeolites for effective conversion of ethylene to propylene. J Ind Eng Chem.

[CR81] Khoshraftar Z, Masoumi H, Ghaemi A (2023). On the performance of perlite as a mineral adsorbent for heavy metals ions and dye removal from industrial wastewater: a review of the state of the art. Case Stud Chem Environ Eng.

[CR82] Khosravi M, Cathey HE, Mackinnon IDR (2021). Comprehensive mineralogical study of Australian zeolites. Microporous Mesoporous Mater.

[CR83] Kilani A, Olubambi A, Ikotun B, Adeleke O, Adetayo O (2022). Structural performance of concrete reinforced with banana and orange peel fibers-a review Journal of Sustainable Construction Materials and Technologies. J Sustain Const Mater Technol.

[CR84] Kneller JM, Pietraß T, Ott KC, Labouriau A (2003). Synthesis of dealuminated zeolites NaY and MOR and characterization by diverse methodologies: 27Al and 29Si MAS NMR, XRD, and temperature dependent 129Xe NMR. Microporous Mesoporous Mater.

[CR85] Kong SH, Chin CYJ, Yek PNY, Wong CC, Wong CS, Cheong KY, Liew RK, Lam SS (2022). Removal of heavy metals using activated carbon from microwave steam activation of palm kernel shell. Environ Adv.

[CR86] Kwon S, Choi Y, Singh BK, Na K (2020). Selective and rapid capture of Sr2+ with LTA zeolites: effect of crystal sizes and mesoporosity. Appl Surf Sci.

[CR87] Lahiri SK, Zhang C, Sillanpää M, Liu L (2022). Nanoporous NiO@SiO2 photo-catalyst prepared by ion-exchange method for fast elimination of reactive dyes from wastewater. Mater Today Chem.

[CR88] Le TM, Nguyen GT, Dat ND, Tran NT (2023). An innovative approach based on microwave radiation for synthesis of zeolite 4A and porosity enhancement. Results Eng.

[CR89] Lee MG, Park JW, Kam SK, Lee CH (2018). Synthesis of Na-A zeolite from Jeju Island scoria using fusion/hydrothermal method. Chemosphere.

[CR90] Lee WH, Lin YW, Lin KL (2022). Parameter optimization, characterization, and crystallization mechanisms underlying the synthesis of zeolite A using liquid crystal display waste glass and sandblasting waste as alternative raw materials. J Environ Chem Eng.

[CR91] Lee JB, Ahmed I, Lee G, Kim TW, Kim CU, Jhung SH (2023). Synthesis of SSZ-13 zeolites using calcined rice husk as silica source for propylene production from ethylene and carbon dioxide adsorption. J Ind Eng Chem.

[CR92] Lee WH, Lin YW, Lin KL (2023). Optimization of synthesis parameters for the preparation of zeolite by reusing industrialwaste as raw material: sandblasting waste and solar panel waste glass. Solid State Sci.

[CR93] Li Y, Li L, Yu J (2017). Applications of zeolites in sustainable chemistry. Chem.

[CR94] Li W, Jin H, Xie H, Ma L (2023). Synthesis of zeolite A and zeolite X from electrolytic manganese residue, its characterization and performance for the removal of Cd2+ from wastewater. Chin J Chem Eng.

[CR95] Li B, Lin X, Zhao Y (2024). Facile preparation and application of magnetic chitosan/fly ash composite as a hybrid biosorbent for the effective removal of direct dyes. J Mol Liq.

[CR96] Li G (2005) FT-IR studies of zeolite materials: characterization and environmental applications. Ph.D. Thesis, Graduate College, The University of Iowa, Iowa City, p 162

[CR97] Lima EC, Hosseini-Bandegharaei A, Moreno-Piraján JC, Anastopoulos I (2019). A critical review of the estimation of the thermodynamic parameters on adsorption equilibria. Wrong use of equilibrium constant in the Van’t Hoof equation for calculation of thermodynamic parameters of adsorption. J Mol Liq.

[CR98] Lin KL, Lee TC, Hwang CL (2015). Effects of sintering temperature on the characteristics of solar panel waste glass in the production of ceramic tiles. J Mater Cycles Waste Manage.

[CR99] Lin L, Lin Y, Li C, Wu D, Kong H (2016). Synthesis of zeolite/hydrous metal oxide composites from coal fly ash as efficient adsorbents for removal of methylene blue from water. Int J Miner Process.

[CR100] Lin YW, Lee WH, Lin KL (2022). A novel approach for preparing ecological zeolite material from solar panel waste lass and sandblasting waste: microscopic characteristics and humidity control performance. J Market Res.

[CR101] Liu B, Zhang L, Ning K, Yang W (2023). Biochar with nanoparticle incorporation and pore engineering enables enhanced heavy metals removal. J Environ Chem Eng.

[CR102] Llovet X (2019) Microscopy | Electron probe microanalysis. Encycl Anal Sci 30–38. 10.1016/B978-0-12-409547-2.14369-0

[CR103] Ma XY, Fan TT, Wang G, Li ZH, Lin JH, Long YZ (2022). High performance GO/MXene/PPS composite filtration membrane for dye wastewater treatment under harsh environmental conditions. Composites Communications.

[CR104] Ma T, Zhang L, Song Y, Shang Y, Zhai Y, Gong Y (2018) A comparative synthesis of ZSM-5 with ethanol or TPABr template: distinction of Brønsted/Lewis acidity ratio and its impact on n-hexane cracking †. Cite This: Catal Sci Technol 8. 10.1039/c7cy02418e

[CR105] Mabuza L, Sonnenberg N, Marx-Pienaar N (2023). Natural versus synthetic dyes: consumers’ understanding of apparel coloration and their willingness to adopt sustainable alternatives. Resour Conserv Recycling Adv.

[CR106] Mamaghani FAA, Salem A, Salem S (2022). Role of aluminum resource in conversion of bentonite into low silica-based zeolites via fusion technology. Mater Lett.

[CR107] Maraddi AS, D’souza GB, Nataraj SK (2024). MOF-based-membranes for dyes removal from wastewater. Ref Module Mater Sci Mater Eng.

[CR108] Mohod CV, Dhote J, Author C, Gadge Baba Amravati University S, Professor A (2013) Review of heavy metals in drinking water and their effect on human health. Int J Innov Res Sci Eng Technol 2. www.ijirset.com

[CR109] Moisés MP, Da Silva CTP, Meneguin JG, Girotto EM, Radovanovic E (2013). Synthesis of zeolite NaA from sugarcane bagasse ash. Mater Lett.

[CR110] Molina A, Poole C (2004). A comparative study using two methods to produce zeolites from fly ash. Miner Eng.

[CR111] Moudar J, El Fami N, Diouri A, Taibi M (2022). Identification and characterization of faujasite zeolite phase in alkali activated class F fly ash. Mater Today Proc.

[CR112] Mu B, Wang A (2016). Adsorption of dyes onto palygorskite and its composites: a review. J Environ Chem Eng.

[CR113] Muraza O, Abdul-Lateef A, Tago T, Nandiyanto ABD, Konno H, Nakasaka Y, Yamani ZH, Masuda T (2015). Microwave-assisted hydrothermal synthesis of submicron ZSM-22 zeolites and their applications in light olefin production. Microporous and Mesoporous Mater.

[CR114] Murukutti MK, Jena H (2022). Synthesis of nano-crystalline zeolite-A and zeolite-X from Indian coal fly ash, its characterization and performance evaluation for the removal of Cs+ and Sr2+ from simulated nuclear waste. J Hazard Mater.

[CR115] Musyoka NM, Petrik LF, Balfour G, Gitari WM, Hums E (2011). Synthesis of hydroxy sodalite from coal fly ash using waste industrial brine solution. J Environ Sci Health Part A.

[CR116] Ndlovu NZN, Ameh AE, Petrik LF, Ojumu TV (2023). Synthesis and characterisation of pure phase ZSM-5 and sodalite zeolites from coal fly ash. Mater Today Commun.

[CR117] Ng EP, Awala H, Tan KH, Adam F, Retoux R, Mintova S (2015). EMT-type zeolite nanocrystals synthesized from rice husk. Microporous and Mesoporous Mater.

[CR118] Ng TYS, Chew TL, Yeong YF (2019). Synthesis of small pore zeolite via ultrasonic-assisted hydrothermal synthesis. Mater Today: Proc.

[CR119] Nnaji PC, Anadebe VC, Ezemagu IG, Onukwuli OD (2022). Potential of Luffa cylindrica seed as coagulation-flocculation (CF) agent for the treatment of dye wastewater: Kinetic, mass transfer, optimization and CF adsorption studies. Arab J Chem.

[CR120] Oliveira JA, Cunha FA, Ruotolo LAM (2019). Synthesis of zeolite from sugarcane bagasse fly ash and its application as a low-cost adsorbent to remove heavy metals. J Clean Prod.

[CR121] Ozaki H, Sharma K, Saktaywin W (2002). Performance of an ultra-low-pressure reverse osmosis membrane (ULPROM) for separating heavy metal: effects of interference parameters. Desalination.

[CR122] Pal D, Sen S (2024). Optimal synthesis of dolochar derived faujasite zeolite X for highly effective Cd(II) removal. Environ Res.

[CR123] Patel MG, Marakana PG, Dey A, Saini B, Chokshi H (2023). Coal fly ash derived adsorbent for enhancing waste water treatment. Mater Today: Proc.

[CR124] Pauletto PS, Gonçalves JO, Pinto LAA, Dotto GL, Salau NPG (2020). Single and competitive dye adsorption onto chitosan–based hybrid hydrogels using artificial neural network modeling. J Colloid Interface Sci.

[CR125] Payá J, Monzó J, Borrachero MV, Soriano L, Akasaki JL, Tashima MM (2017) New inorganic binders containing ashes from agricultural wastes. Sustain Nonconventional Const Mater Using Inorg Bonded Fiber Compos 127–164. 10.1016/B978-0-08-102001-2.00006-1

[CR126] Payá J, Monzó J, Borrachero MV, Tashima MM, Soriano L (2018) Bagasse ash. Waste and supplementary cementitious materials in concrete: characterisation, properties and applications 559–598. 10.1016/B978-0-08-102156-9.00017-1

[CR127] Pazouki G, Tao Z, Saeed N, Kang WH (2023). Using artificial intelligence methods to predict the compressive strength of concrete containing sugarcane bagasse ash. Constr Build Mater.

[CR128] Pedebos MES, Druzian DM, Oviedo LR, Ruiz YPM, Galembeck A, Pavoski G, Espinosa DCR, da Silva WL (2024). Removal of Rhodamine B dye by adsorption onto an eco-friendly zeolite and machine learning modeling. J Photochem Photobiol, A.

[CR129] Pérez-Botella E, Valencia S, Rey F (2022). Zeolites in Adsorption Processes: State of the Art and Future Prospects.

[CR130] Phi Long B, Van Thiet D, Phi Hung P, Hoang Tuan N, Huu Bac L (2024). Green synthesis of NiTiO3 nanoparticles and sonocatalytic degradation of Rhodamine B textile dye in water. Mater Lett.

[CR131] Plazzotta S, Manzocco L, Nicoli MC (2017). Fruit and vegetable waste management and the challenge of fresh-cut salad. Trends Food Sci Technol.

[CR132] Prajaputra V, Abidin Z, Widiatmaka Suryaningtyas DT, Rizal H (2019) Characterization of Na-P1 zeolite synthesized from pumice as low-cost materials and its ability for methylene blue adsorption. IOP Conf Ser: Earth Environ Sci 399(1). 10.1088/1755-1315/399/1/012014

[CR133] Prasad DS, Sanjana B, Sai Kiran D, Srinivasa Kumar PP, Ratheesh R (2023). A unique sustainable chemical method for the recovery of pure silicon from waste crystalline silicon solar panels. Sustain Mater Technol.

[CR134] Ragheb E, Shamsipur M, Jalali F, Mousavi F (2022). Modified magnetic-metal organic framework as a green and efficient adsorbent for removal of heavy metals. J Environ Chem Eng.

[CR135] Rao F, Liu M, Liu C, Deng W, Huang R, Liao C, Qi T, Hu G (2024). Synthesis of binder-free pelletized Y zeolite for CO2 capture. Carbon Capture Science & Technology.

[CR136] Ren X, Liu S, Qu R, Xiao L, Hu P, Song H, Wu W, Zheng C, Wu X, Gao X (2020). Synthesis and characterization of single-phase submicron zeolite Y from coal fly ash and its potential application for acetone adsorption. Microporous Mesoporous Mater.

[CR137] Ren Z, Wang L, Li Y, Zha J, Tian G, Wang F, Zhang H, Liang J (2022). Synthesis of zeolites by in-situ conversion of geopolymers and their performance of heavy metal ion removal in wastewater:a review. J Clean Prod.

[CR138] Rodríguez‐Couto S, Osma JF, Toca‐Herrera JL (2009). Removal of synthetic dyes by an eco-friendly strategy. Eng Life Sci.

[CR139] San Martin D, Ramos S, Zufía J (2016). Valorisation of food waste to produce new raw materials for animal feed. Food Chem.

[CR140] Santasnachok C, Kurniawan W, Hinode H (2015). The use of synthesized zeolites from power plant rice husk ash obtained from Thailand as adsorbent for cadmium contamination removal from zinc mining. J Environ Chem Eng.

[CR141] Sayehi M, Delahay G, Tounsi H (2022). Synthesis and characterization of ecofriendly materials zeolite from waste glass and aluminum scraps using the hydrothermal technique. J Environ Chem Eng.

[CR142] Serati-Nouri H, Jafari A, Roshangar L, Dadashpour M, Pilehvar-Soltanahmadi Y, Zarghami N (2020). Biomedical applications of zeolite-based materials: a review. Mater Sci Eng, C.

[CR143] Shahnaz A, Shahzadi P (2016) Utilization of bio materials as pozzolanic material for partial replacement of Cement. J Chem Mater Res 5(5):85–91. www.oricpub.com

[CR144] Sharma HB, Vanapalli KR, Barnwal VK, Dubey B, Bhattacharya J (2021). Evaluation of heavy metal leaching under simulated disposal conditions and formulation of strategies for handling solar panel waste. Sci Total Environ.

[CR145] Shen F, Yuan H, Pang Y, Chen S, Zhu B, Zou D, Liu Y, Ma J, Yu L, Li X (2013). Performances of anaerobic co-digestion of fruit & vegetable waste (FVW) and food waste (FW): single-phase vs. two-phase. Biores Technol.

[CR146] Shi L, Wang Q, Zhao X, Che Y, Liu H, Zuo W, Zhang Y (2023). The methyl blue adsorption performance and mechanism of NaX zeolite synthesized from Huadian oil shale ash. J Taiwan Inst Chem Eng.

[CR147] Shu R, Qiao Q, Guo F, Dong K, Liu S, Xu L, Bai Y, Zhou N (2023). Controlled design of Na–P1 zeolite/ porous carbon composites from coal gasification fine slag for high-performance adsorbent. Environ Res.

[CR148] Shubair T, Eljamal O, Tahara A, Sugihara Y, Matsunaga N (2019). Preparation of new magnetic zeolite nanocomposites for removal of strontium from polluted waters. J Mol Liq.

[CR149] Siddique R, Kunal, Mehta A (2020) Utilization of industrial by-products and natural ashes in mortar and concrete development of sustainable construction materials. Nonconventional Vernac Constr Mater: Characterisation Prop Appl 247–303. 10.1016/B978-0-08-102704-2.00011-1

[CR150] Singh B (2018) Rice husk ash. Waste and supplementary cementitious materials in concrete: characterisation, properties and applications 417–460. 10.1016/B978-0-08-102156-9.00013-4

[CR151] Sivalingam S, Sen S (2018). Optimization of synthesis parameters and characterization of coal fly ash derived microporous zeolite X. Appl Surf Sci.

[CR152] Skotta A, Jmiai A, Elhayaoui W, El-Asri A, Tamimi M, Assabbane A, El Issami S (2023). Suspended matter and heavy metals (Cu and Zn) removal from water by coagulation/flocculation process using a new bio-flocculant: Lepidium sativum. J Taiwan Inst Chem Eng.

[CR153] Somerset VS, Petrik LF, White RA, Klink MJ, Key D, Iwuoha E (2004). The use of X-ray fluorescence (XRF) analysis in predicting the alkaline hydrothermal conversion of fly ash precipitates into zeolites. Talanta.

[CR154] Somsiripan T, Sangwichien C (2023). Enhancement of adsorption capacity of methylene blue, malachite green, and Rhodamine B onto KOH activated carbon derived from oil palm empty fruit bunches. Arab J Chem.

[CR155] Soomro M, Tam VWY, Jorge Evangelista AC (2023) Industrial and agro-waste materials for use in recycled concrete. Recycled Concrete: Technol Perform 47–117. 10.1016/B978-0-323-85210-4.00009-6

[CR156] Stepanov AG (2016) Basics of solid-state NMR for application in zeolite science: material and reaction characterization. Zeolites Zeolite-like Mater 137–188. 10.1016/B978-0-444-63506-8.00004-5

[CR157] Suárez M (2001) Capítulo cinco. Negociando con el rey. Desafíos Transatlánticos 255–314. 10.4000/BOOKS.IFEA.4052

[CR158] Supelano GI, Gómez Cuaspud JA, Moreno-Aldana LC, Ortiz C, Trujillo CA, Palacio CA, Parra Vargas CA, Mejía Gómez JA (2020). Synthesis of magnetic zeolites from recycled fly ash for adsorption of methylene blue. Fuel.

[CR159] Suresh Kumar BV (2007) Characterization of zeolites by infrared spectroscopy. Art Asian J Chem. https://www.researchgate.net/publication/283862901

[CR160] Tabassum N, Rafique U, Ashraf MA (2018). Novel method for doping of vanadium into zeolites synthesized from industrial refused materials and application for environmental remediation. Ekoloji.

[CR161] Tan WC, Yap SY, Matsumoto A, Othman R, Yeoh FY (2011). Synthesis and characterization of zeolites NaA and NaY from rice husk ash. Adsorption.

[CR162] Tao Z, Tian Y, Ou SY, Gu Q, Shang J (2023). Direct air capture of CO2 by metal cation-exchanged LTA zeolites: effect of the charge-to-size ratio of cations. AIChE J.

[CR163] Tauanov Z, Shah D, Inglezakis V, Jamwal PK (2018). Hydrothermal synthesis of zeolite production from coal fly ash: a heuristic approach and its optimization for system identification of conversion. J Clean Prod.

[CR164] Technischen Fakultät D, Toniolo N (2019) Novel geopolymers incorporating silicate waste Neuartige Geopolymere aus silikatischen Industrieabfällen

[CR165] Tokay B, Akpınar I (2021). A comparative study of heavy metals removal using agricultural waste biosorbents. Bioresource Technology Reports.

[CR166] Tran-Nguyen PL, Ly KP, Thanh LHV, Angkawijaya AE, Santoso SP, Tran NPD, Tsai ML, Ju YH (2021). Facile synthesis of zeolite NaX using rice husk ash without pretreatment. J Taiwan Inst Chem Eng.

[CR167] Trueman CN, Rodgers KJ, McLellan IS, Hursthouse AS (2019) Geochemistry | Inorganic. Encycl Anal Sci 271–282. 10.1016/B978-0-12-409547-2.14362-8

[CR168] Truttim P, Asavapisit S, Piyaphanuwat R (2023) Microwave synthesis of zeolite X from bituminous fly ash and its characterization. Mater Today: Proceedings. 10.1016/J.MATPR.2023.06.023

[CR169] Velarde L, Sadegh Nabavi M, Escalera E, Antti M-L, Akhtar F (2023) Adsorption of heavy metals on natural zeolites: a review. Chemosphere 328:138508. 10.1016/j.chemosphere.2023.138508 10.1016/j.chemosphere.2023.138508 36972873

[CR170] Velusamy K, Periyasamy S, Kumar PS, Jayaraj T, Krishnasamy R, Sindhu J, Sneka D, Subhashini B, Vo DVN (2021). Analysis on the removal of emerging contaminant from aqueous solution using biochar derived from soap nut seeds. Environ Pollut.

[CR171] Velusamy K, Periyasamy S, Kumar PS, Rangasamy G, Nisha Pauline JM, Ramaraju P, Mohanasundaram S, Nguyen Vo DV (2022). Biosensor for heavy metals detection in wastewater: a review. Food Chem Toxicol.

[CR172] Vijay Ramteke A, Mishra D, Mishra S, Pant KK, Bhatia D (2023). Conversion of fly-ash into pristine ZSM-5 and its application for methane dehydroaromatization reaction. J Ind Eng Chem.

[CR173] Visa M (2016). Synthesis and characterization of new zeolite materials obtained from fly ash for heavy metals removal in advanced wastewater treatment. Powder Technol.

[CR174] Volkov DS, Rogova OB, Proskurnin MA (2021) Organic matter and mineral composition of silicate soils: FTIR comparison study by photoacoustic, diffuse reflectance, and attenuated total reflection modalities. Agron 11(9):1879. 10.3390/AGRONOMY11091879/S1 10.3390/nano10122501 PMC7764527 33322144

[CR175] Wang M, Xu D, Ma H, Li B, Howard A (2023). Synthesis of NaA zeolite from foundry dust and its adsorption capacity of ammonia. J Environ Manage.

[CR176] Wang YS, Huo TR, Wang Y, Bai JW, Huang PP, Li C, Deng SY, Mei H, Qian J, Zhang XC, Ding C, Zhang QY, Wang WK (2024). Constructing mesoporous biochar derived from waste carton: improving multi-site adsorption of dye wastewater and investigating mechanism. Environ Res.

[CR177] Xiang L, Guo Z, Yang L, Qin Y, Chen Z, Wang T, Sun W, Wang C (2023). Modification of crystal growth of NaA zeolite with steric hindrance agents for removing ammonium ion from aqueous solution. J Ind Eng Chem.

[CR178] Yang L, Jiang T, Xiong P, Yang S, Gao M, Nagasaka T (2023). Green activating silica-alumina insoluble phase of fly ash to synthesize zeolite P with high adsorption capacity for Pb(II) in solution. Adv Powder Technol.

[CR179] Yeung KL, Han W (2014). Zeolites and mesoporous materials in fuel cell applications. Catalysis Today.

[CR180] Yin X, Liu N, Han M, Xu F, Jia Y, Song F, Cui H (2023). Ultrasonic-pretreated hydrothermal synthesis of less dense zeolite CHA from the transformation of zeolite T. Ultrason Sonochem.

[CR181] Yuan N, Tan K, Zhang X, Zhao A, Guo R (2022). Synthesis and adsorption performance of ultra-low silica-to-alumina ratio and hierarchical porous ZSM-5 zeolites prepared from coal gasification fine slag. Chemosphere.

[CR182] Yuan N, Zhao A, Hu Z, Tan K, Zhang J (2022). Preparation and application of porous materials from coal gasification slag for wastewater treatment: a review. Chemosphere.

[CR183] Zhang M, Zhang H, Xu D, Han L, Niu D, Tian B, Zhang J, Zhang L, Wu W (2011). Removal of ammonium from aqueous solutions using zeolite synthesized from fly ash by a fusion method. Desalination.

[CR184] Zhang T, Wang W, Zhao Y, Bai H, Wen T, Kang S, Song G, Song S, Komarneni S (2021). Removal of heavy metals and dyes by clay-based adsorbents: from natural clays to 1D and 2D nano-composites. Chem Eng J.

[CR185] Zhang X, Li C, Zheng S, Di Y, Sun Z, Zhang X, Li C, Zheng S, Di Y, Sun Z (2022). A review of the synthesis and application of zeolites from coal-based solid wastes. Int J Miner Metall Mater.

[CR186] Zhang L, Zhang K, Kang S, He S, Dong X, Zhao Y, Li F, Cen Q (2023). Study on using crystalline celluloses as templates for preparation of hierarchical LTA zeolite for fast Cd(II) removal. Chem Eng Res Des.

[CR187] Zhao C, Ge R, Zhen Y, Wang Y, Li Z, Shi Y, Chen X (2019). A hybrid process of coprecipitation-induced crystallization-capacitive deionization-ion exchange process for heavy metals removal from hypersaline ternary precursor wastewater. Chem Eng J.

[CR188] Zhao Y, Lai GS, Li C, Wang R (2023). Acid-resistant polyamine hollow fiber nanofiltration membrane for selective separation of heavy metals and phosphorus. Chem Eng J.

[CR189] Zheng M, Sun Z, Han H, Zhang Z, Ma W, Xu C (2021). Enhanced coagulation coupled with heavy metal capturing for heavy metals removal from coal gasification brine and a novel mathematical model. J Water Proc Eng.

[CR190] Zhou J, Zheng F, Li H, Wang J, Bu N, Hu P, Gao JM, Zhen Q, Bashir S, LouiseLiu J (2020). Optimization of post-treatment variables to produce hierarchical porous zeolites from coal gangue to enhance adsorption performance. Chem Eng J.

[CR191] Zhou Q, Jiang X, Qiu Q, Zhao Y, Long L (2023). Synthesis of high-quality NaP1 zeolite from municipal solid waste incineration fly ash by microwave-assisted hydrothermal method and its adsorption capacity. Sci Total Environ.

[CR192] Zijun Z, Effeney G, Millar GJ, Stephen M (2021). Synthesis and cation exchange capacity of zeolite W from ultra-fine natural zeolite waste. Environ Technol Innov.

